# Multimaterial Shape Memory Polymer Fibers for Advanced Drug Release Applications

**DOI:** 10.1007/s42765-025-00571-4

**Published:** 2025-06-18

**Authors:** Xue Wan, Siyao Chen, Jingqi Ma, Chaoqun Dong, Hritwick Banerjee, Stella Laperrousaz, Pierre-Luc Piveteau, Yan Meng, Jinsong Leng, Fabien Sorin

**Affiliations:** 1 Institute of Materials, École Polytechnique Fédérale de Lausanne, 1015 Lausanne, Switzerland; 2 Centre for Composite Materials and Structures, Harbin Institute of Technology, Harbin, 150080 People’s Republic of China; 3 Guangzhou Institute of Advanced Technology, Guangzhou, 511458 People’s Republic of China

**Keywords:** Shape memory polymers, Multimaterial fibers, Drug delivery, Sequential drug release, Multifunctionality

## Abstract

Stimuli-responsive polymers offer unprecedented control over drug release in implantable delivery systems. Shape memory polymer fibers (SMPFs), with their large specific surface area and programmable properties, present promising alternatives for triggerable drug delivery. However, the existing SMPFs face limitations in resolution, architecture, scalability, and functionality. We introduce thermal drawing as a materials and processing platform to fabricate microstructured, multimaterial SMPFs that are tens of meters long, with high resolution (10 μm) and extreme aspect ratios (> 10^5^). These novel fibers achieve highly controlled, sequential drug release over tailored time periods of 6 months. Post thermal drawing photothermal coatings enable accelerated, spatially precise drug release within 4 months and facilitate light-triggered, untethered shape recovery. The fibers’ fast self-tightening capability within 40 s shows their potential as smart sutures for minimally invasive procedures that deliver drugs simultaneously. In addition, the advanced multimaterial platform facilitates the integration of optical and metallic elements within SMP systems, allowing highly integrated fibers with shape memory attributes and unprecedented functionalities. This versatile technology opens new avenues for diverse biomedical applications, including implantable drug delivery systems, smart sutures, wound dressings, stents, and functional textiles. It represents a significant advancement in precise spatio-temporal control of drug delivery and adaptive medical devices.

**Graphical Abstract:**

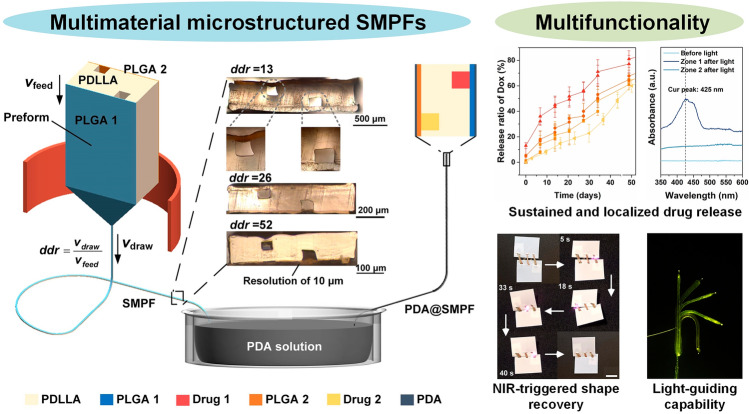

**Supplementary Information:**

The online version contains supplementary material available at 10.1007/s42765-025-00571-4.

## Introduction

Implantable drug delivery systems have revolutionized therapeutic treatments by offering spatio-temporal controlled release, significantly minimizing side effects and drug resistance compared to traditional methods such as oral, intravenous injection, or topical administration [1–3]. Stimuli-responsive polymers have advanced these systems, enabling drug release triggered by external stimuli, such as light, electric, or magnetic fields [4–6]. Among these materials, shape memory polymers (SMPs) stand out due to their ability to recover their original shape upon stimulation, combined with biocompatibility and biodegradability, making them ideal for minimally invasive, triggerable drug delivery applications [7–11]. Shape memory polymer fibers (SMPFs), in particular, offer a high specific surface area, large aspect ratio, and light weight, allowing for enhanced drug loading capacity and controlled release kinetics [12–14]. Their ease of processing into fibrous structures, such as membranes, scaffolds, or textiles, makes them highly adaptable for biomedical engineering applications [15–17].

Despite their promise however, the existing SMPFs still face many challenges in resolution, architecture, scalability, and functionality. Common techniques for producing SMPFs include electrospinning [18, 19], melt spinning [20], and wet spinning [21]. Although electrospun SMPFs demonstrate high resolution, their small diameters make them more suited to fiber-assembly types of architectures, such as tissues where high mechanical strength is not required. Melt-spun SMPFs exhibit longer lengths and high yields, but the high temperature required to spin at low viscosity may be detrimental for many drugs. Moreover, melt and wet spinning typically allow for simple fiber architectures where only a single drug can be uniformly dispersed throughout the fiber, with the release rate primarily dictated by the degradation speed of the host SMP. This restricts independent control over drug release and biodegradability, hindering the development of complex drug delivery systems that require sequential and precise spatio-temporal control of multiple drugs for synergistic treatments. In addition, most SMPFs respond only to temperature changes, limiting their utility in applications requiring remote, noninvasive actuation. Light-responsive SMPs offer a solution, enabling untethered and safe actuation with highly precise control [22].

Recent advances in the thermal drawing technique, a multimaterial fiber processing platform, have been paving the way for the integration of several advanced functionalities in a single fiber [23–27]. This technique involves assembling various materials, including metals, polymers, insulators, and semiconductors, into a macroscopic preform, which is then heated and co-drawn into a long, continuous fiber at the softening temperature [28–30]. This technique can produce kilometer-long fibers while preserving the intricate cross-sectional architecture of the preform, achieving sizes from millimeters to micrometers. These multifunctional thermally drawn fibers have potential applications across biomedical engineering [31, 32], electronics [33–35], photonic devices [30, 36, 37], and robotics [38, 39]. Thus far, however, thermally drawn SMPFs have been seldom investigated [40], and complete fibers with shape memory capabilities combined with other important functionalities such as controlled drug release, electrical conductivity, or optical wave guiding, remain to be demonstrated.

Here, we propose a novel strategy that leverages the attributes of thermal drawing to fabricate tens-of-meters-long, uniform, microstructured, multimaterial SMPFs with a micro-meter scale resolution and extreme aspect ratios greater than 10^5^. These fibers can incorporate several drug reservoirs, each sealed by biodegradable polymers with different degradation rates, allowing for independent control over sequential, sustained drug release while maintaining the long-term stability of the SMP matrix. Photothermal coatings further enhance this system by enabling light-triggered, accelerated, and precisely controlled spatio-temporal release of multiple drugs. The SMPFs also exhibit a self-tightening behavior under light, showing promise as smart sutures for minimally invasive surgery. In addition, we integrate metallic and optical components within the SMPFs, which brings an unprecedented level of multifunctionality to SMP systems. We believe that these multifunctional SMPFs hold great promise for various biomedical devices, including implantable drug delivery systems, advanced sutures, wound dressings, stents, and smart textiles.

## Experimental Section

### Materials

Poly(d,l-lactide acid) (PDLLA) and poly(d,l-lactide-co-glycolic acid) (PLGA) 7507 (PLGA2) were purchased from Corbion Purac. PLGA 504H (PLGA1) was purchased from Evonik Röhm GmbH. PLGA1 has a lactic acid (LA) to glycolic acid (GA) ratio of 75:25 and an inherent viscosity of 0.45–0.60 dl g^−1^, while PLGA2 has a 50:50 ratio and inherent viscosity of 0.7 dl g^−1^. 3D-printed polyethylene terephthalate glycol (PETG) and poly (L-lactic acid) (PLLA) filaments were purchased from Formfutura. Phosphate buffer saline (PBS) pellets (pH: 7.4) were purchased from Thermo Fisher. Tween 80 and Tris Pufferan were purchased from Carl Roth GmbH. Dopamine hydrochloride and curcumin (Cur) were bought from Sigma-Aldrich. Doxorubicin hydrochloride (Dox) was purchased from abcr GmbH. All the polymers were stored at 25 °C under vacuum before use.

### Rheological Characterization

Rheological tests were conducted on compression-molded 1-mm-thick disks of PDLLA, PLGA, and PLLA using an AR 2000ex rheometer (TA Instruments). The viscosity was characterized by an oscillatory temperature ramp from 50 to 250 °C at 3 °C min^−1^, a frequency of 1 Hz, and a strain of 1%. Parallel ETC aluminum plates with a diameter of 25 mm and a Peltier plate were used. Frequency sweep experiments were performed from 10^−4^ to 10^0^ s^−1^ at 140 °C with a strain amplitude of 0.1%. For dynamic oscillation stress sweeps, the stress amplitude was increased from 10^1^ to 10^4^ Pa at 140 °C with a fixed angular frequency of 1 rad s^−1^.

### Thermal Drawing of SMPFs

Polymer granules were placed in a custom compression mold at around 150 °C with adjustable pressure to achieve the desired thickness. The PDLLA–PLGA preform was fabricated by shaping each part through compression molding or mechanical machining, followed by assembly and final consolidation. Custom polytetrafluoroethylene (PTFE) or steel rods were used to create the hollow or core channels. The preform was drawn in a custom drawing tower with top, middle, and bottom temperature at 80, 160, and 60 °C, respectively.

For the PETG–PLGA preform, a 3D-printed PETG core replaced the PDLLA core. The preform was drawn with top, middle, and bottom temperature at 80, 170, and 80 °C, respectively. For metallic wire-integrated SMP preform, stainless steel wire (316 L, McMaster) was introduced during the drawing process, with the same temperature profile as the PDLLA preform.

### Preparation of Polydopamine Nanoparticles on SMPFs

A Tris–HCl solution (pH: 8.5) was prepared from Tris Pufferan. Dopamine hydrochloride was then dissolved in the Tris–HCl solution at concentrations of 2, 4, and 6 mg mL^−1^ and maintained at 40 °C for 72 h. SMPFs were immersed in this solution and subsequently rinsed with deionized water to obtain the polydopamine (PDA)-coated fibers (PDA@SMPF). The size distribution of PDA nanoparticles was analyzed by measuring at least 100 randomly selected nanoparticles from scanning electron microscopy (SEM) images. The photothermal effect of the PDA coatings was monitored using a thermocouple over multiple cycles of near-infrared (NIR) light on and off. The photostability of the PDA particles was assessed by Ultraviolet–Visible (UV–Vis) spectroscopy.

### Drug Release Characterization

Drug solutions were prepared by dissolving Dox or Cur in PBS buffer with 0.5% (v/v) Tween 80 at a concentration of 50 mg mL^−1^. Drug solutions were injected into the fiber’s hollow channels by immersing one end in the solution and applying vacuum at the other end to draw the solution in. After injection, the fiber ends were sealed with water-resistant glue (Araldite Rapid). The drug-loaded SMPFs were immersed in PBS buffer at 37 °C for several months. Drug release was monitored at preset intervals by UV–Vis spectroscopy, measuring Dox and Cur absorbance at 480 and 425 nm, respectively.

### General Characterization

The cross sections of SMPFs were observed using Leica DM 2700 Optical Microscope. Biodegradability was monitored with Gemini SEM 300 operating at 0.5 kV. Thermal properties were analyzed by differential scanning calorimetry (DSC) from 20 to 100 °C at a rate of 10 °C min^−1^ utilizing the second heating cycle. Molecular weights of the polymers were determined by gel permeation chromatography. Thermomechanical properties were measured using dynamic mechanical analysis (DMA) Q800 in tension mode, at 1 Hz frequency, 0.01 N preload, 20 μm amplitude, and 3 °C min^−1^ heating rate. Rectangular samples were cut from compression-molded films, and mechanical properties were evaluated by uniaxial tensile testing (Zwick/Roell with 50 N load cell) at 5 mm min^−1^.

### Shape Memory Characterization

Shape memory cycle (SMC) was performed using DMA Q800 [41, 42]. The procedures are as follows: the sample was heated above *T*_g_ to obtain the initial strain (*ε*_initial_). It was then isothermally stretched to a fixed stress, recording the deformed strain (*ε*_deform_). Next, the sample was cooled below *T*_g_, and the external force was unloaded to obtain the fixed strain (*ε*_fix_). Finally, the sample was reheated above *T*_g_, and the residual strain after recovery was recorded as *ε*_recover_. Shape fixity ratio (*R*_f_) and shape recovery ratio (*R*_r_) were calculated as follows:

1 $$R_{{\text{f}}} = \frac{{\varepsilon_{{{\text{fix}}}} }}{{\varepsilon_{{{\text{deform}}}} }} \times 100\%$$

2 $$R_{{\text{r}}} = \frac{{\varepsilon_{{{\text{deform}}}} - \varepsilon_{{{\text{recover}}}} }}{{\varepsilon_{{{\text{deform}}}} - \varepsilon_{{{\text{initial}}}} }} \times 100\% .$$

### Cytocompatibility Characterization

Primary human dermal fibroblasts (HDF) were chosen for cell viability and proliferation studies. The cells were cultured and maintained in high glucose DMEM (Dulbecco’s modified Eagle’s medium, Gibco, Thermo Fisher Scientific, USA) supplemented with 10% (v/v) fetal bovine serum (FBS) and 1% antibiotic–antimycotic mixture (penicillin 10 units/mL, streptomycin and amphotericin 0.25 μg/mL). The viability of the HDF cells seeded on both the SMPF and PDA@SMPF samples was visualized using 1 μg/mL Calcein-AM and 1 μg/mL Propidium iodide (PI). Samples were conditioned in complete high glucose DMEM (supplemented with 10% FBS) prior cell seeding for 24 h. HDF cells at a density of 1 × 10^4^ cells were seeded on the SMPF and PDA@SMPF, then maintained for 24 h in a humified CO_2_ incubator at 37 °C. Post 1, 2, and 5 days of culture, the HDF cells seeded on the samples were stained with calcein-AM and PI at 37 °C for 15 min and observed under excitation wavelengths of 495 and 535 nm, respectively, after washing with PBS. In the case of live cells, calcein-AM diffuses through the intact membrane and is enzymatically hydrolyzed, giving rise to a uniform green fluorescence. While in the case of dead cells, PI passes through the damaged cell membrane of the dead cells and binds with the nucleic acids to give rise to a bright red fluorescence.

The SMPF and PDA@SMPF samples were immersed in DMEM medium for 24 h. After immersion, the samples were placed into culture wells containing HDF cells at a concentration of 1 × 10^4^ cells/mL. After incubation for 1, 2, and 5 days, cell viability was assessed using the CCK-8 assay. Culture medium containing 10% CCK-8 solution was added to each well, and the plates were incubated at 37 °C for 3 h. The absorbance of the medium was then measured at a wavelength of 450 nm. The absorbance of the medium without any sample was defined as 100%, and the relative cell viability of the samples was calculated accordingly.

### Multifunctionality Characterization

The resistance changes of the metallic wire-integrated SMPF during shape memory cycles were monitored by a multimeter (Keithley 2400). The light transmission performance of the optical fiber-integrated SMPF during shape memory cycles were measured by a radiometer (Thorlabs PM100D).

## Results and Discussion

### Thermal Drawing of the Multimaterial SMPFs

**Fig. 1:**
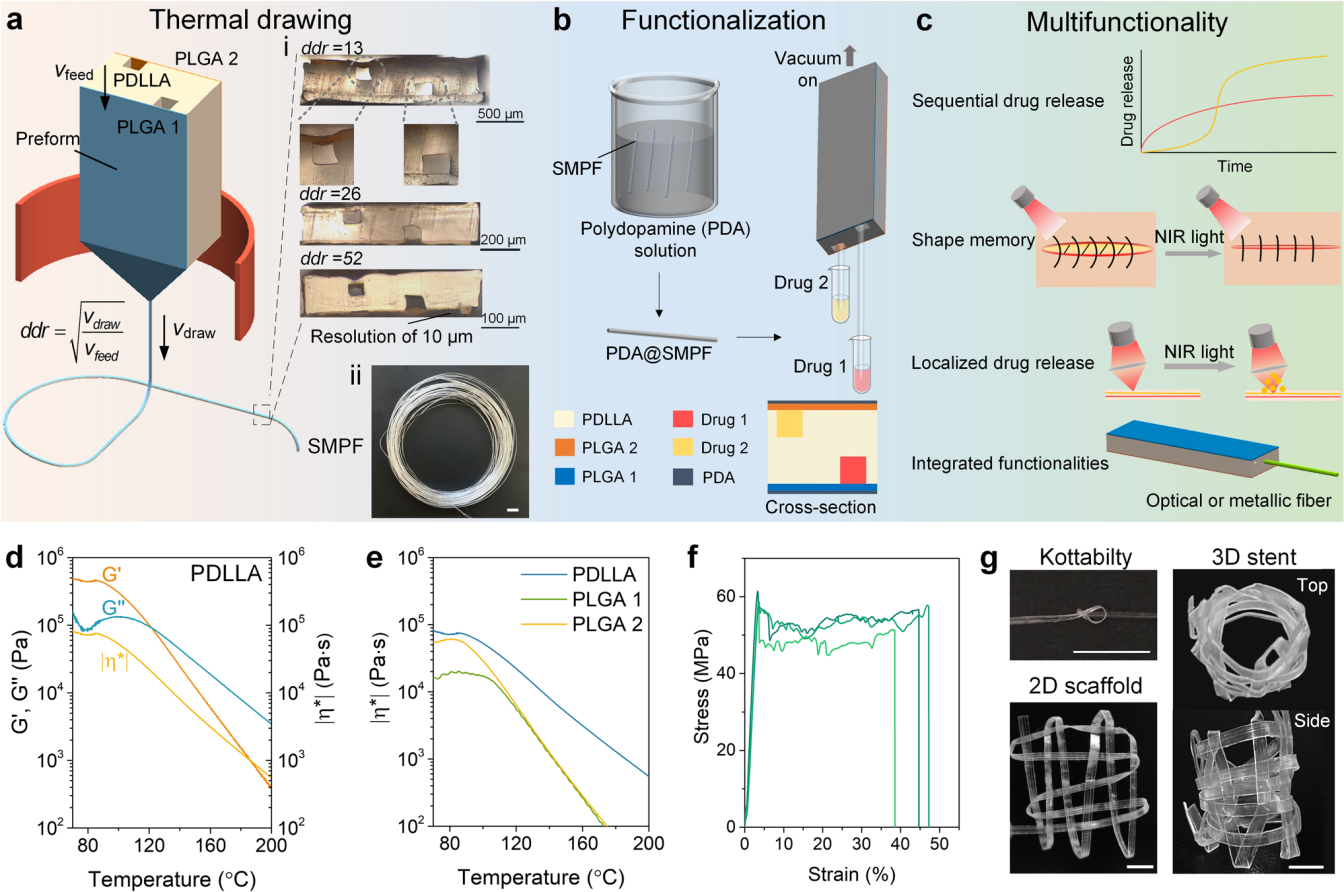
Microstructured, multimaterial SMPF manufacturing. **a** Schematic thermal drawing fabrication. The inset depicts optical cross-sectional images of the thermally drawn SMPF at different *ddr* conditions (i) and a 50-m-long continuous fiber (ii). Scale bar in (ii), 1 cm. **b** Post-fabrication of photothermal PDA coatings and infusion of various drugs. **c** Schematic multifunctionality of the SMPF. **d** Oscillatory shear rheological properties of PDLLA as a function of temperature. **e** Complex viscosity comparison between PDLLA and PLGA variants. **f** Static tensile curves of the SMPF at room temperature. The curves correspond to multiple fiber samples. **g** Images of a knot, a woven 2D scaffold, and a 3D stent. Scale bars, 5 mm

We selected PDLLA and two types of PLGA to create a novel drug delivery system. PDLLA serves as the fiber matrix due to its excellent shape memory properties and long-term stability, while the PLGA variants enable sequential drug release, with PLGA1 degrading faster than PLGA2. Our innovative fabrication process, illustrated in Fig. 1a, involves thermally drawing a preform comprising PDLLA with two hollow channels sealed by PLGA films serving as drug reservoirs. This method preserves the preform’s multimaterial structure while reducing fiber size according to the draw–down ratio, *ddr* = (*v*_draw_/*v*_feed_)^1/2^, where *v*_draw_ and *v*_feed_ are the drawing and feeding speeds, respectively, resulting in an increase in fiber length due to volume conservation. After thermal drawing, the fibers are coated with PDA nanoparticles, enhancing their responsiveness to NIR light [43]. In addition, various drugs are infused into the hollow channels using a vacuum pump, as shown in Fig. 1b. The resulting multimaterial PDA@SMPF exhibits unprecedented multifunctionality, including sequential drug release, on-demand shape recovery, localized drug release, and seamless integration with optical or metallic fibers as we will show below and exemplified in Fig. 1c. This multifaceted approach marks a significant leap forward in advanced complex drug delivery systems, offering independent and precise spatio-temporal control over multiple drug release while maintaining structural integrity and adaptability.

The shear rheological properties of amorphous PDLLA were first investigated to understand its suitability with thermal drawing. Upon heating, it transitioned from an elastic to a viscous phase at 122 °C, where the loss modulus (*G*″) dominated over the storage modulus (*G*′) (Fig. 1d). Above this temperature, the moduli decreased gradually, enabling the stable thermal drawing of PDLLA into continuous fibers [30]. In contrast, semi-crystalline PLLA (Fig. S1) showed a rapid drop in moduli above the crossover point, making stable thermal drawing difficult. Both the PLGA variants exhibited gradual transitions, with moduli intersections at 100 and 101 °C (Fig. S2). PDLLA had a higher complex viscosity (|η^*^|) than both PLGA1 and PLGA2, and their similar decreasing trends upon heating met the requirement for co-thermal drawing (Fig. 1e). Shear-thinning behavior and high apparent yield stress, typical in thermal drawing, were also observed (Fig. S3). In contrast, when PDLLA was substituted by PETG in a similar preform structure, the PLGA barriers within the drawn fiber collapsed due to a temperature mismatch (Fig. S4).

The fiber resolution was tailored via *ddr*, with both the fiber size and PLGA film thickness decreasing as *ddr* increased. Figure 1a(i) shows multiple cross-sections of microstructured SMPFs. The preform architecture was maintained at the fiber level. The thickness of the sealed PLGA films decreased from 100 to 10 μm as *ddr* increased from 13 to 52. Additionally, the two channels encapsulated by the PLGA films were well preserved and in particular did not collapse. Consequently, 50-m-long, continuous multimaterial SMPFs with a high resolution of 10 μm and an extreme aspect ratio of 10^5^ were successfully fabricated [Fig. 1a(ii)]. Regarding their mechanical properties, the microstructured SMPFs exhibit an elastic modulus of (2301.3 ± 257.9) MPa, a tensile strength of (58.9 ± 2.4) MPa, and an elongation at break of (43.5 ± 4.5)% (Figs. 1f and S5). In comparison, the compression-molded PDLLA and PLGA variants demonstrated an elastic modulus of 1300–1800 MPa, a tensile strength of 40–50 MPa, and an elongation at break of 3–8% (Table S1). The superior strength and elongation at break of the SMPF can be attributed to the high orientation of molecular chains during fabrication. The SMPF demonstrated good knotting and weaving abilities into a 2D scaffold and a 3D stent (Fig. 1g), highlighting great potential for stimuli-responsive textiles.

### Sequential and Sustained Release of Multiple Drugs from SMPFs

**Fig. 2:**
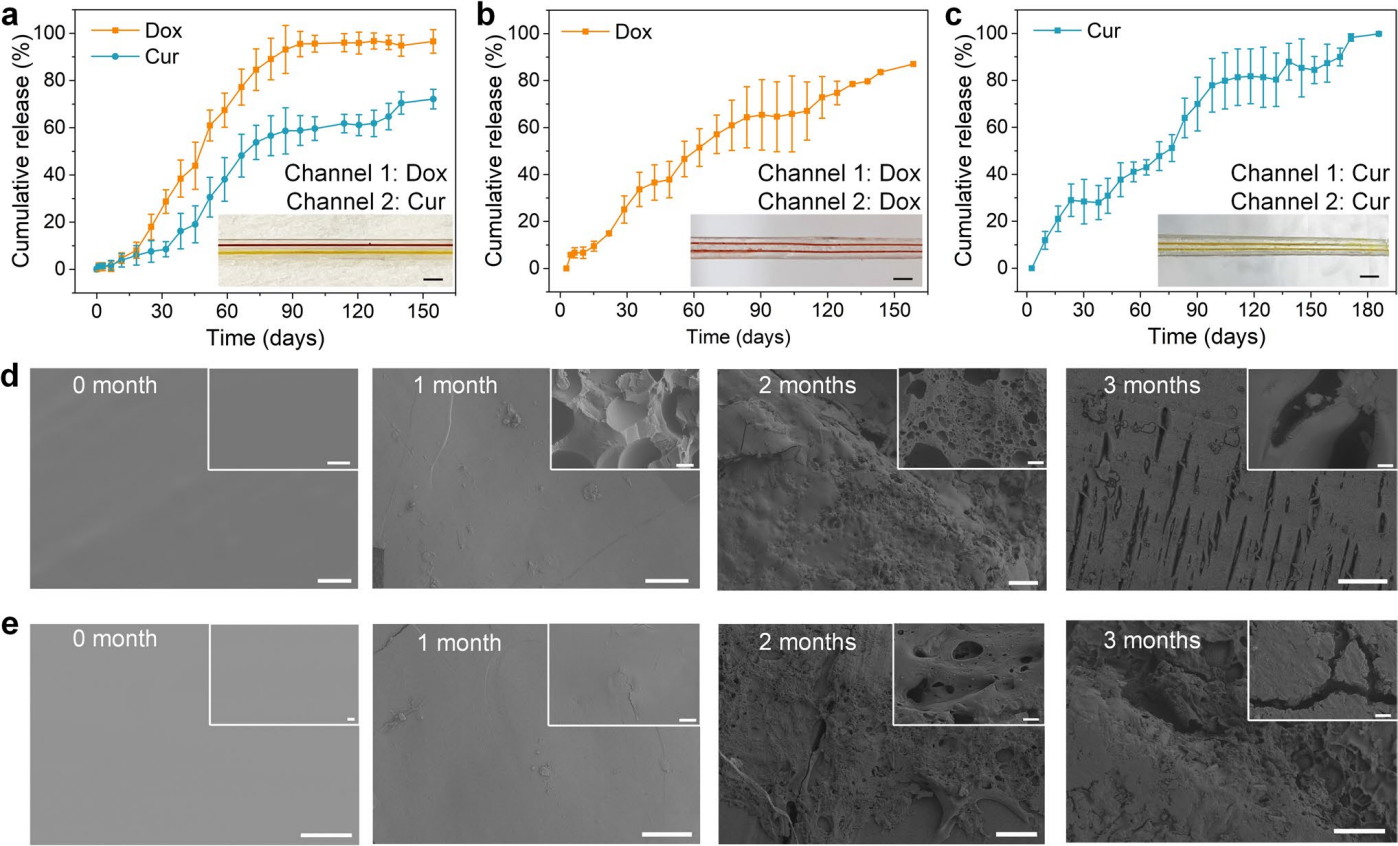
Sustained multiple drug release and morphological evolution of the SMPFs. **a** Release profiles from the SMPF with PLGA1 encapsulating Dox and PLGA2 encapsulating Cur. **b** Release profiles from the SMPF with both PLGA variants encapsulating Dox. **c** Release profiles from the SMPF with both PLGA variants encapsulating Cur. Inset pictures in **a**–**c** show the respective SMPFs loaded with different drugs (Scale bars, 1 mm). Morphological changes of **d** PLGA1 and **e** PLGA2 during the degradation periods at 1, 2, and 3 months. Scale bars, 100 μm (Inset: 2 μm)

We selected Dox, a chemotherapy agent, and Cur, an anticancer drug, for co-delivery as an effective synergistic treatment strategy to overcome multidrug resistance and adverse reactions associated with individual chemotherapeutic agents [44]. The two separate hollow channels within the SMPF were employed to independently load the drugs, each sealed with a distinct PLGA film (PLGA1 or PLGA2). The film thickness was controlled at around 50 μm by adjusting *ddr*. The drug loading process (Fig. S6) ensured precise filling of each channel with the designated drug solution and achieved a loading efficiency above 95% (Supporting Information), which is comparable to drug-loading efficiencies reported for polymer fiber systems fabricated by other techniques, typically in the range of 76–100% [45–47]. Figure 2a shows the sequential drug release evolution from the SMPF, with PLGA1 and PLGA2 encapsulating the Dox and Cur reservoirs, respectively (Fig. 2a inset). Real-time drug release data were obtained based on the calibration curves of the two drug solutions (Figs. S7–S8). The release profiles of Dox and Cur exhibit different time-dependent rates. In the first 30 days, Dox was released faster than Cur, consistent with PLGA1’s shorter degradation time compared to PLGA2. From days 30–60, the release accelerated, following the established kinetics of PLGA-controlled delivery systems, initially dominated by diffusion and later accelerated by the formation of water-filled pores as erosion progressed [48]. The drug release profiles were in good agreement with the empirical Weibull mathematical model (Sect. S6 and Table S2). After 90 days, Dox release approached nearly 100% and plateaued as PLGA1 completely degraded, while Cur continued to release.

Figure 2b shows the drug release evolution from the SMPF, with both PLGA1 and PLGA2 encapsulating the Dox reservoirs (Fig. 2b inset). Throughout the release period, the release velocity remained constant initially, dominated by the faster degradation of PLGA1. After plateauing from days 30–45, the release speed increased due to the delayed degradation of PLGA2. Detailed changes in release rates will be discussed in the morphological evolution of the PLGA films later. This time-dependent release behavior aligns perfectly with our design concept of sequentially controlled drug release by adjusting the SMPF compositions. These results highlight the potential for precise temporal control over multiple drug release, yielding synergistic treatment effects in a programmed time sequence. Figure 2c illustrates the drug release evolution from the SMPF, with both PLGA1 and PLGA2 encapsulating the Cur reservoirs (Fig. 2c inset), indicating sustained and complete drug release over 6 months.

The morphological changes of the PLGA films were monitored during degradation and are shown in Fig. 2d, e. Initially, both films had smooth surfaces without observable pores. After 1 month, PLGA1 developed loose holes and small cracks, while PLGA2 remained intact, aligning with the initial release profile in Fig. 2a. As degradation continued, cracks in PLGA1 propagated, creating diffusion pathways for the resulting oligomers and micromolecules. In contrast, PLGA2 began to decompose and form holes due to prolonged interaction with PBS, accelerating its release rate. After 3 months, PLGA1 exhibited significant swelling and erosion, resulting in numerous large holes and gaps, while PLGA2’s holes were less interconnected, preventing oligomers from escaping and resulting in a lower release rate. The morphology of PDLLA (Fig. S9) remained intact throughout the release period, indicating its low degradation rate and stable performance compared to the degrading PLGA films. Fig. S10 shows that both the number average molecular weight (*M*_n_) and the weight average molecular weight (*M*_w_) of the PLGA variants significantly decreased, while PDLLA experienced only a slight reduction. This demonstrates that the drug release behavior was primarily governed by the independent degradation of the PLGA variants, while the SMP matrix maintained its structural integrity.

### Photothermal Effect of PDA@SMPFs

**Fig. 3:**
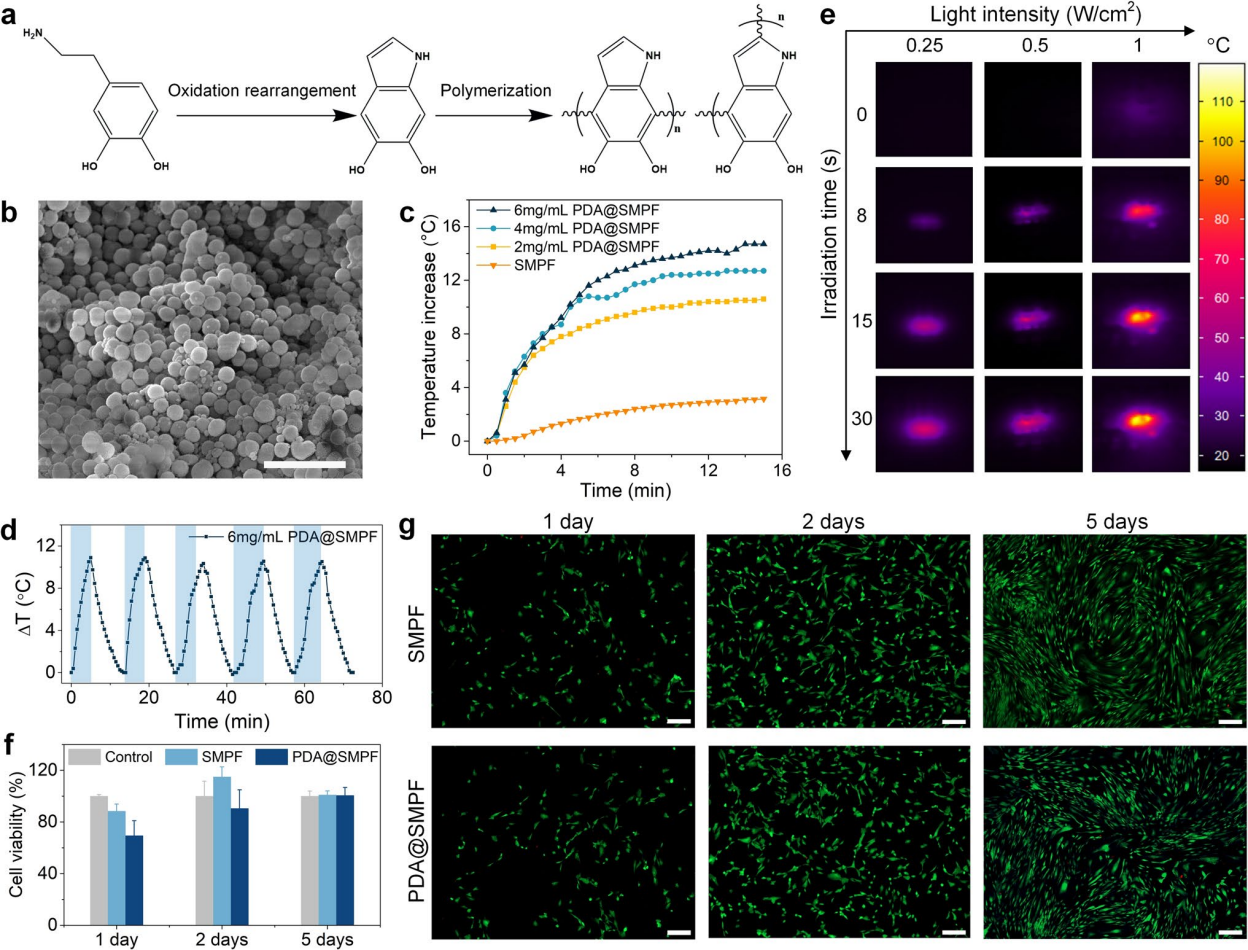
Photothermal conversion and stability of the PDA@SMPFs. **a** Schematic of PDA nanoparticle synthesis. **b** Morphology of the PDA nanoparticles. Scale bar, 1 μm. **c** NIR light-triggered photothermal curves of the PDA@SMPFs with varying PDA concentrations in PBS buffer (light intensity: 1 W cm^−2^). **d** Photothermal response of the PDA@SMPF with a PDA concentration of 6 mg mL^−1^ over five cycles of laser on (blue background) and off. **e** Real-time thermal images of the PDA@SMPF at a PDA concentration of 6 mg mL^−1^ in air. **f** Cell viability of HDF cells cultured for 1, 2, and 5 days on the SMPF and PDA@SMPF. **g** Fluorescence image of HDF cells with the SMPF and PDA@SMPF in 5 days. Green and red colors correspond to live and dead cells, respectively. Scale bars, 250 µm

We now turn to investigating a higher level of integration by functionalizing the SMPF with a photothermal PDA coating to allow for on-demand and spatially resolved drug release. Figure 3a illustrates the self-polymerization of PDA coatings. PDA nanoparticles, synthesized using a dopamine (DA) concentration of 6 mg mL^−1^ in Tris–HCl, exhibited a spherical shape with an average diameter of (546 ± 109) nm (Figs. 3b and S11a). Under NIR light irradiation, PDA@SMPF immersed in PBS converted photon energy into heat, raising the buffer temperature (Fig. 3c). Increasing the concentration of PDA nanoparticles from 2 to 6 mg mL^−1^ enhanced photothermal efficiency, resulting in a maximum temperature increase of ~ 15 °C, as compared to ~ 3 °C in uncoated fibers. The photostability test (Fig. 3d) demonstrates consistent photothermal conversion across five cycles of laser on (5 min) and off (8 min), confirming stable heat generation.

Under NIR light in air, thermal imaging (Fig. 3e) clearly captured the light intensity- and irradiation time-dependent photothermal effects. The temperature rose rapidly and reached 100 °C, hence above the glass transition temperature (*T*_g_) of PDLLA (Fig. S12 and Table S3). This demonstrates the high photothermal conversion efficiency of PDA coatings under NIR light, both in PBS buffer and air, making them suitable for NIR light-triggered shape recovery. The absorbance of the PDA@SMPFs remained stable over 50 min of irradiation (Fig. S11b), indicating remarkable photostability. These attributes make the fibers promising for targeted and localized photothermal therapy.

### Cytocompatibility Assessment

In order to use these fibers for medical implantable device, a primary requirement must be non-cytotoxic. Herein, the HDF cells were cultured to evaluate the cytocompatibility of the specimens. Figure 3f shows the influence of SMPF and PDA@SMPF on cell viability within 3 days. It can be clearly found that the cell viability for SMPF was more than 88% on the first day. After co-culture for  2 and 5 days, the cell viability was more than 100%. Although the cell viability for PDA@SMPF was only around 70% on the first day, it increased to more than 90% after 2 and 5 days. The LIVE/DEAD Viability/Cytotoxicity Kit was also employed to observe the morphology of HDF cells. Most of the detected cells were live as indicated by the green color, with very few dead cells detected in both the SMPF and PDA@SMPF within 5 days (Fig. 3g). The cells exhibited a spindle-shaped morphology over time, suggesting good cell viability and adhesion. These observations indicate that both the SMPF and PDA@SMPF have no apparent cytotoxicity and could be potentially used as biomaterials.

### Spatio-Temporal Control of Multiple Drug Release from PDA@SMPFs

**Fig. 4:**
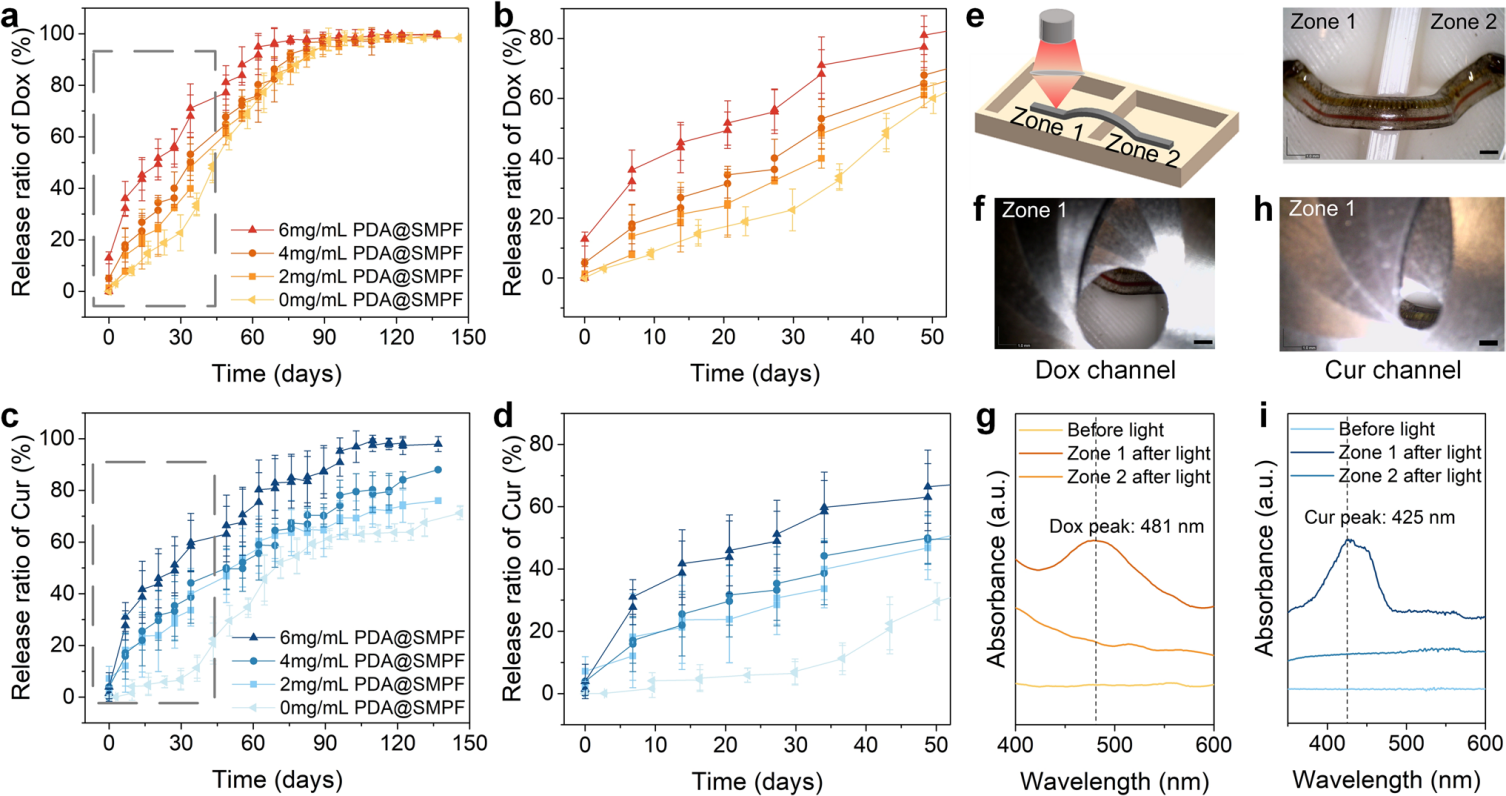
Light-triggered spatio-temporal control of multiple drug release from the PDA@SMPFs with PLGA1 and PLGA2 encapsulating Dox and Cur, respectively. **a** Release ratio of Dox. **b** Enlarged view of Dox release. **c** Release ratio of Cur. **d** Enlarged view of Cur release. **e** Schematic and experimental setup for spatially resolved drug release. **f**–**g** Local Dox release when NIR light irradiates the channel sealed by the PLGA1 film. **h**–**i** Local Cur release when NIR light irradiates the channel sealed by the PLGA2 film. Scale bars, 1 mm

Local drug release under intermittent NIR light irradiation, applied weekly for 10 min, was investigated on the PDA@SMPFs with PLGA1 and PLGA2 encapsulating Dox and Cur reservoirs, respectively. Figure 4a depicts the light-triggered drug release profile of Dox from the fibers with varying PDA coating concentrations (0–6 mg mL^−1^). The high photothermal efficiency of the PDA nanoparticles induced a rapid temperature increase, leading to an immediate Dox release. The elevated temperature enhanced the hydrolysis of ester bonds in the PLGA polymer backbone, leading to accelerated degradation. As degradation progressed, holes appeared on the surface of the PLGA films, which further promoted drug release [49]. In addition, the increased temperature enhanced drug diffusion through the PLGA films, contributing to a more pronounced burst release. The PDA@SMPFs with a PDA concentration of 6 mg mL^−1^ shortened the release period to 2 months, 1 month earlier than the SMPF without NIR irradiation (Fig. S13). The magnified view of the initial release periods (Fig. 4b) reveals a sudden increase in drug release immediately after NIR irradiation, which was absent in the SMPFs. In addition, higher PDA concentrations accelerated the release rate, resulting in an earlier plateau in the Dox release curve.

The release profile of Cur exhibited a similar trend (Fig. 4c). Although the SMPF required nearly 6 months for complete Cur release (Fig. 2c), the PDA coatings significantly accelerated the release rate, reducing the release period. As shown in Fig. 4d, the Cur release ratio from the PDA@SMPF with a 6 mg mL^−1^ PDA concentration was nearly three times greater than that of the SMPF. The triggering of the PDA coating response after 3 months indicate good long-term stability of the coating, as also shown in several studies [49–51]. The photothermal coatings, combined with NIR irradiation, enabled substantial modulation and control over the release speed and duration. To exclude the effect of NIR irradiation alone, drug release profiles of the SMPF were compared with and without intermittent NIR irradiation (Fig. S14), revealing no significant differences.

Subsequently, spatially resolved drug release at specific locations was investigated. An adjustable iris was placed between the NIR light source and the PDA@SMPF to precisely control the transmitted light intensity and irradiated area (Fig. S15). The PDA@SMPF was positioned in a self-made two-zone device containing PBS buffer, allowing for controlled single drug release at a specific location within Zone 1 (Fig. 4e). By tuning the iris aperture, NIR light was sequentially directed at the permeable reservoir channels loaded with Dox and Cur, enabling independent control of their release. Figure 4f, g show a sudden increase in absorbance at 481 nm, while Fig. 4h, i show a similar increase at 425 nm, corresponding to the characteristic peaks of Dox and Cur, respectively. In contrast, neither Zone 1 before light irradiation nor Zone 2 after irradiation exhibited any peak signals, demonstrating precise spatial control over the targeted drug release under NIR light.

### Light-Triggered Shape Recovery of PDA@SMPFs

**Fig. 5:**
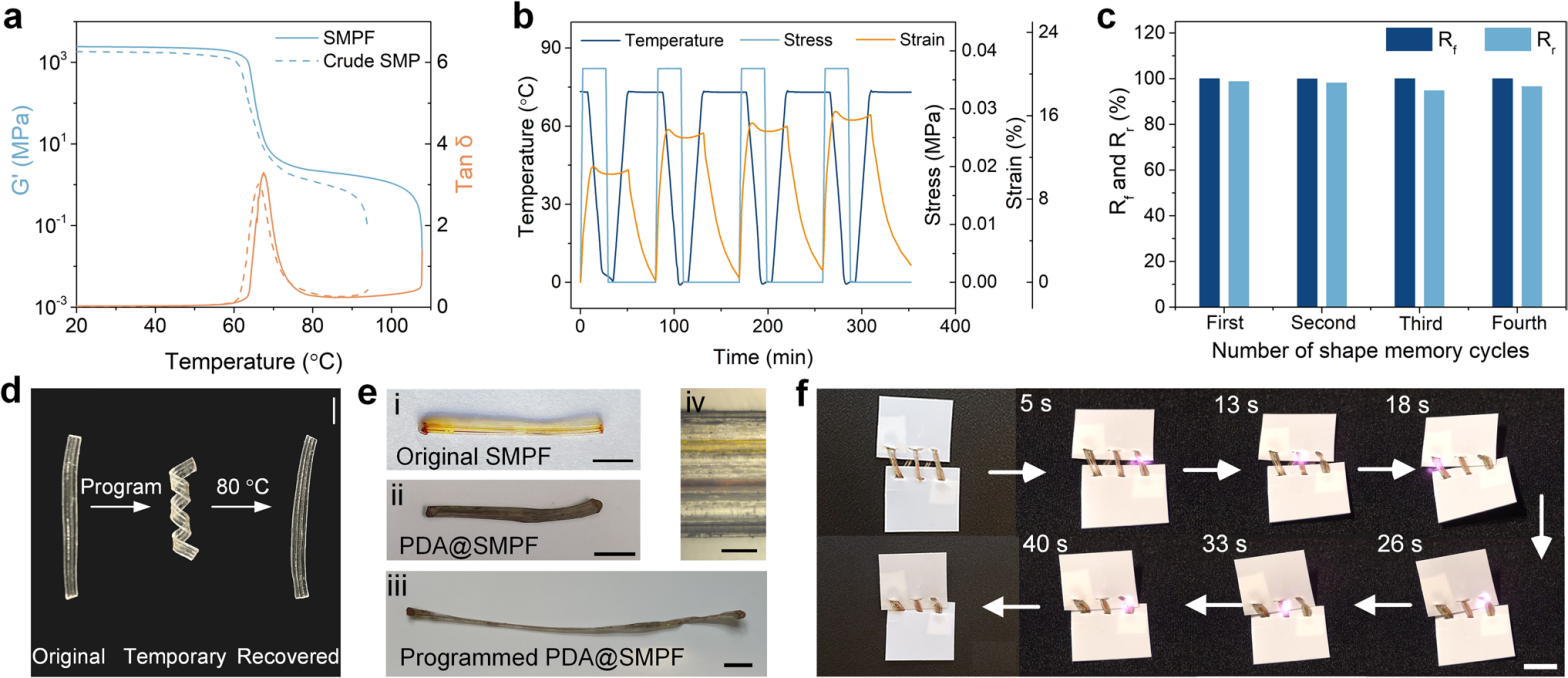
Shape memory properties of the multimaterial SMPF and PDA@SMPF. **a** Comparison of *G*′ and tan *δ* between the SMPF and compression-molded SMP film. **b** Shape memory cycles at a recovery temperature of 80 °C. **c** Quantitative shape memory properties. **d** Shape programming and recovery of a spiral fiber at 80 °C. Scale bar, 5 mm. **e** Shape programming of the PDA@SMPF into an elongated temporary shape. Scale bars, 1 cm for (i)–(iii), 200 μm for (iv). **f** Self-tightening behavior of the PDA@SMPF closing a gap between two films under NIR light (light intensity: 1 W cm^−2^). Scale bar, 1 cm

We now turn to demonstrating the versatility of our approach by showing the shape memory properties that the fibers exhibit in addition to precise spatio-temporal control of multiple drug release. As the fiber matrix material, the thermomechanical properties of PDLLA after thermal drawing were first investigated. The peak of the damping factor (tan *δ*), corresponding to *T*_g_, shows a slight increase compared to the material of the fiber before drawing (Fig. 5a). This increase can be attributed to the orientation of polymer molecules along the drawing direction during fabrication [52]. The *G*′ increased from 1830 to 2420 MPa in the glassy state and from 1.2 to 2.3 MPa in the rubbery state. The DSC curve reveals a *T*_g_ of 50.2 °C for PDLLA, slightly higher than that of the PLGA variants (Fig. S12). The higher *T*_g_ observed in DMA compared to DSC is due to the hysteresis effect in DMA, where polymer chains experience delayed relaxation under dynamic loading during testing [53]. The thermomechanical properties of the PLGA variants are shown in Fig. S16 and Table S2.

Analysis of the temperature-dependent thermoviscoelastic behavior of PDLLA (Fig. S17) reveals a decrease in relaxation time with increasing temperature. By following the relaxation time at *σ*/*σ*_0_ = e^−1^, where *σ* and *σ*_0_ are the real-time and original relaxation moduli, respectively, the activation energy (*E*_a_) of PDLLA was derived from the Arrhenius equation, yielding a value of 265.8 kJ mol^−1^ (Fig. S18 and Sect. S11). In addition, the as-fabricated SMPFs exhibited an actuation force of 1.18 N and an actuation stress of 3.81 MPa due to the high orientation of molecular chains during thermal drawing process, which can be released through heating (Fig. S19). These temperature-dependent stress relaxation properties enable the fibers to fully relax while maintaining their drawn size.

We studied the shape memory properties of the microstructured SMPF through SMC at 80 °C (Fig. 5b). *R*_r_ and *R*_f_ are 98.7% and 99.9%, respectively (Fig. 5c), indicating excellent shape memory performance. These properties can be flexibly adjusted by modifying the test conditions. For instance, at 68 °C, *R*_f_ increased to 99.9%, while *R*_r_ decreased to 85.3% during the second SMC (Fig. S20). In addition, immersing the SMPF in a water bath at 80 °C triggered rapid recovery from a temporary spiral shape to a flat shape within 17 s (Figs. 5d and S21, and Movie S1). The recovery force was determined by DMA by fixing the strain of the programmed stretched SMPF during heating. The maximum recovery force and stress reached 0.5 N and 1.7 MPa, respectively (Fig. S22).

The photothermal effect of PDA coatings endowed the SMPFs with light-triggered shape memory attributes. The PDA@SMPF, loaded with Dox and Cur, was programmed into an elongated shape and sewn between two PTFE films, leaving a gap (Fig. 5e). NIR light stimulation induced the shape recovery of the fiber, closing the gap within 40 s (Fig. 5f and Movie S2). This self-tightening behavior was also verified under heat stimulus in a water bath at 55 °C, achieving complete recovery within 3 s (Fig. S23 and Movie S3). The shorter shape recovery time in water was attributed to the uniform heating process across the entire structure. Noncontact NIR stimulation has been proven safe and nontoxic for cells [54, 55], allowing for precise and localized control. The shape memory capability of the PDA@SMPF presents significant potential as a biodegradable self-tightening suture for minimally invasive surgery, while achieving sequential and precise spatio-temporal control of multiple drug release under NIR light.

### Integrated Multifunctionality of SMPFs

**Fig. 6:**
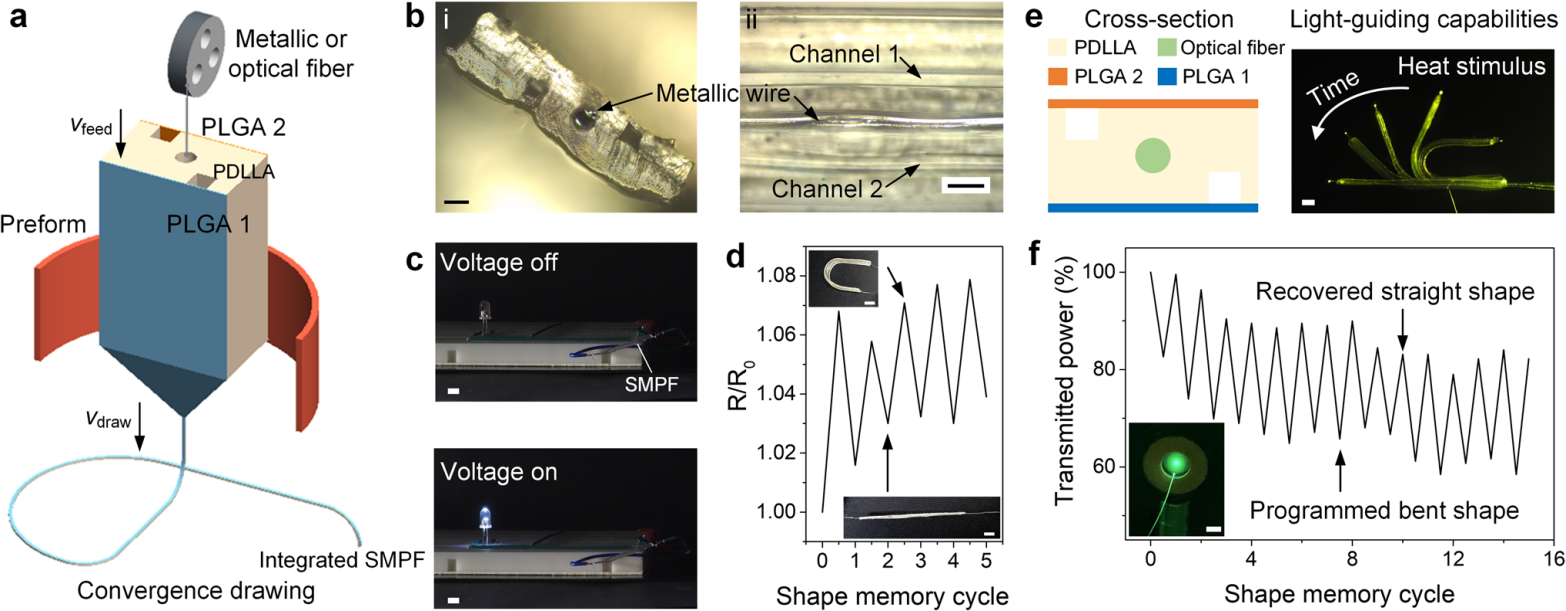
Integration of functional elements within SMPFs. **a** Schematic convergence drawing process of a fiber wire-integrated SMPF. **b** Cross-sectional and side-view images of a metallic wire-integrated SMPF. Scale bars: left, 100 μm; right, 200 μm. **c** Application of a metallic wire-integrated SMPF as an electrical component to trigger an LED light using a 3 V power source. Scale bars, 5 mm. **d** Relative resistance change of the metallic wire-integrated SMPF during five shape memory cycles. Inset images show the SMPF in the temporary bent and recovered straight states. Scale bars, 5 mm. **e** Schematic cross-section of an optical fiber-integrated SMPF and the merged shape recovery process at 80 ℃ while guiding green light. Scale bar, 2 mm. **f** Light transmission performance of the optical fiber-integrated SMPF during fifteen shape memory cycles. Inset image illustrates the detection of output light power. Scale bar, 5 mm

Finally, we leverage the unique attributes of the thermal drawing process by integrating multiple materials and functional elements within the SMP and drug releasing fibers. Figure 6a depicts the schematic thermal drawing process, where a fiber wire element is introduced into the central channel of an SMP preform. This approach, known as convergence drawing [56], integrates a solid and continuous metallic (or optical fiber) wire into the SMPFs within a drawn polymer fiber. The cross-sectional and side-view images of a meters-long, stainless steel wire-integrated SMP fiber are shown in Fig. 6b and S24. The integrated metallic wire added high electrical conductivity to the fiber so that it could serve as an electrical component to trigger an LED light using a 3 V power source (Fig. 6c and S25). The resistance exhibited a slight increase in the temporary bent shape and decreased in the recovered flat shape (Fig. 6d). The calculated electrical conductivities before and after five cycles were 1.16 × 10^6^ and 1.15 × 10^6^ S m^−1^, respectively, indicating stable and robust electrical performance throughout shape memory process. To the best of our knowledge, this is the first time that an SMP is thermally drawn within a microstructured multimaterial fiber incorporating metallic materials allowing for such advanced and diverse functionalities.

Additionally, the hollow channel in the SMPF can also integrate optical fibers. Figure 6e illustrates that the optical fiber-integrated SMPF maintained its light-emitting function, guiding green light, while seamlessly recovering its original shape at 80 °C. The light transmission performance during fifteen shape memory cycles is shown in Fig. 6f. The transmitted light power decreased in the programmed bent state and partially recovered after returning to the flat shape. Over multiple cycles, a gradual decline in overall transmissions was observed, which was in agreement with the previous findings [40]. This may be attributed to the micro damage accumulated during repeated deformation and recovery. Nevertheless, the shape memory property enables adaptive and programmable tuning of output light power, showing potential for reconfigurable optical device capable of switching between different transmission states. The integration of optical fibers enhances the functionality of SMPFs, enabling light transmission during localized drug release and shape recovery under light stimulus. This allows for the monitoring at the drug delivery site and potentially improving the therapeutic efficacy of advanced drug delivery systems.

**Fig. 7:**
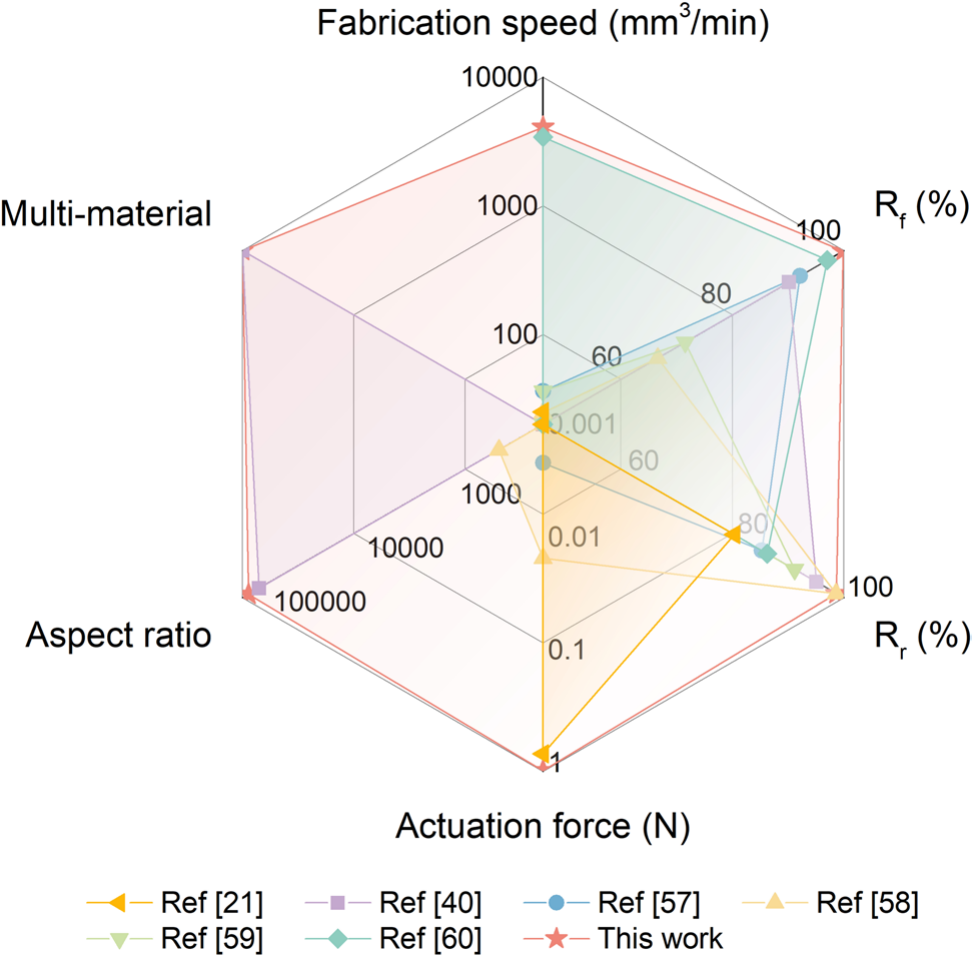
Rader plot of three actuation parameters and three processing parameters compared to reported SMP fibers

Figure 7 provides a radar chart for the overall performance evaluation of three actuation parameters and three processing parameters compared to reported SMP fibers [21, 40, 57–60]. The SMPFs in this work are capable of balancing high performance in all six criteria, including high fabrication speed (4080 mm^3^ min^−1^), excellent shape memory properties (nearly 100%), extreme aspect ratios (> 10^5^), relatively high actuation force (1.18 N), and multimaterial integration to realize multifunctionality. These capabilities would broaden the application of smart fibers toward advanced drug release control, as well as electronics and photonic devices.

In summary, we utilize thermal drawing to fabricate multimaterial SMPFs with complex architectures, precise spatio-temporal control over multiple drug release, and unprecedented integrated multifunctionality. When compared with nanoparticle and hydrogel-based drug delivery systems, SMPFs offer superior long-term stability, mechanical properties, and programmable, localized drug release. SMPFs can sustain release for up to 6 months, outperforming nanoparticles and hydrogels that degrade relatively quickly and lack mechanical strength, making SMPFs ideal for long-term, load-bearing applications. The thermal drawing technique enables scalable production with high resolution, addressing the batch-to-batch variability and scalability constraints common in conventional systems. It is worth mentioning that although two specific PLGA types were selected in this study, the degradation rate can be tuned from 2 weeks to over a year by selecting appropriate monomer ratios and molecular weights, providing flexibility for a variety of clinical applications. Additionally, SMPFs can integrate metallic and optical components for multifunctionality, and the combined shape memory behavior further holds great promise for minimally invasive surgical applications. These capabilities are rarely achievable in conventional drug delivery systems.

The multifunctionality of SMPFs offers broad opportunities for clinical applications. Their high specific surface area and precise spatio-temporal control of drug delivery makes them highly suitable for implantable systems targeting chronic conditions. The self-tightening behavior of SMPFs opens up opportunities for smart sutures capable of both tissue repair and localized delivery of multiple drugs with synergistic effects, potentially reducing repeated administrations and enhancing patient comfort. The integration of photothermal coatings enables photothermal therapy (PTT) for targeted ablation of tumor cells while minimizing damage to normal tissues [61]. Such a synergistic approach could improve therapeutic efficacy while reducing systemic side effects compared to chemotherapy or PTT alone. Additionally, the embedded conductive elements within the fibers hold great promise for alternative electric field-triggered release. The combination of shape memory behavior and light-guiding capabilities make SMPFs strong candidates for advanced endoscopic probes in vascular or catheter procedures requiring dexterous navigation [40]. The large scalability supports the transition from 1D fibers to 3D biomedical implants, such as wound dressings, stents, and wearable therapeutic textiles through knitting and weaving.

Although these prospects are promising, several challenges remain to be addressed. Future research should focus on improving large-scale manufacturing efficiency while reducing costs. For instance, larger-diameter preforms and higher drawing speeds would significantly reduce fabrication time and costs [62]. Secondary morphologies such as grooved fibers can be explored through thermal drawing by designing optimized preform structures that can promote directed cell growth and water regulation [63]. Additionally, although preliminary in vitro cytotoxicity assays have been performed and all employed polymers are FDA-approved biocompatible materials, further biomedical validation is essential. Future efforts should include long-term in vivo studies to assess biocompatibility, particularly regarding immune or inflammatory responses and the potential effects of polymer degradation byproducts on surrounding tissues. The combined therapeutic efficacy for enhanced cancer therapy by implanting the fibers into solid tumors in animal models also needs to be investigated [64]. This requires comprehensive assessments of antitumor activity, biodistribution, and overall therapeutic outcomes [65]. Such investigations are critical for establishing biosafety and demonstrating translational potential in preclinical applications.

## Conclusions

In this work, we demonstrated thermally drawn multimaterial SMPFs as advanced drug delivery systems that provide precise spatio-temporal control over multiple drug release for synergistic treatment. These fibers feature a high resolution of 10 μm, a uniform diameter, and an extreme aspect ratio of 10^5^. By incorporating two drug reservoirs for Dox and Cur, sealed with biodegradable polymers that exhibit different degradation rates, we achieved independent temporal control over sequential and sustained drug release for up to 6 months while ensuring the long-term stability of the SMP matrix. Post thermal drawing, the SMPFs were coated with photothermal PDA nanoparticles, enabling accelerated drug release under intermittent NIR irradiation, reducing the releasing duration to 4 months, and facilitating spatially resolved drug delivery to targeted positions. Additionally, the PDA coatings endow the SMPFs with light-triggered, untethered, on-demand shape recovery with precise control. The rapid self-tightening behavior of drug-loaded fibers within 40 s underscores their potential as smart sutures for minimally invasive surgery with concurrent drug delivery. Finally, we leveraged our multimaterial processing platform to integrate metallic and optical components within the SMPFs, bringing unprecedented multifunctionality. The integration of optical fibers allows continuous light transmission throughout the shape recovery process, enhancing the potential for monitoring the drug delivery sites and improving therapeutic efficacy. This versatile technology for fabricating multimaterial SMPs can offer novel opportunities for implantable drug delivery systems, wound dressings, stents, smart textiles, and adaptive medical devices.

## Supplementary Information

Below is the link to the electronic supplementary material.

Supplementary file 1 (DOCX 2814 KB)

Supplementary file 2 (MP4 20948 KB)

Supplementary file 3 (MP4 27538 KB)

Supplementary file 4 (MP4 6711 KB)

## Data Availability

The data are available from the corresponding author on reasonable request.
