# Supplementary material for: Multimaterial Shape Memory Polymer Fibers for Advanced Drug Release Applications
Source: Adv Fiber Mater. 2025 Jun 18;7(5):1576–89. doi: 10.1007/s42765-025-00571-4 (PMC12420707; doi:10.1007/s42765-025-00571-4)
Supplement: Supplementary file 1 — (DOCX 2814 KB) [file 42765_2025_571_MOESM1_ESM.docx]

**Multi-Material Shape Memory Polymer Fibers for Advanced Drug Release Applications**

Xue Wan,^1,2^ Siyao Chen,^2^ Jingqi Ma,^3^ Chaoqun Dong,^1^ Hritwick Banerjee,^1^ Stella Laperrousaz,^1^ Pierre-Luc Piveteau,^1^ Yan Meng,^1^ Jinsong Leng,^2^ and Fabien Sorin^1,^[[1]](#footnote-1)^*^

^1^ Institute of Materials, École Polytechnique Fédérale de Lausanne, 1015 Lausanne, Switzerland

^2^ Centre for Composite Materials and Structures, Harbin Institute of Technology, Harbin, 150080, PR China

^3^ Guangzhou Institute of Advanced Technology, Guangzhou, 511458, PR China

**Section S1. Rheological properties for thermal drawing.**

**
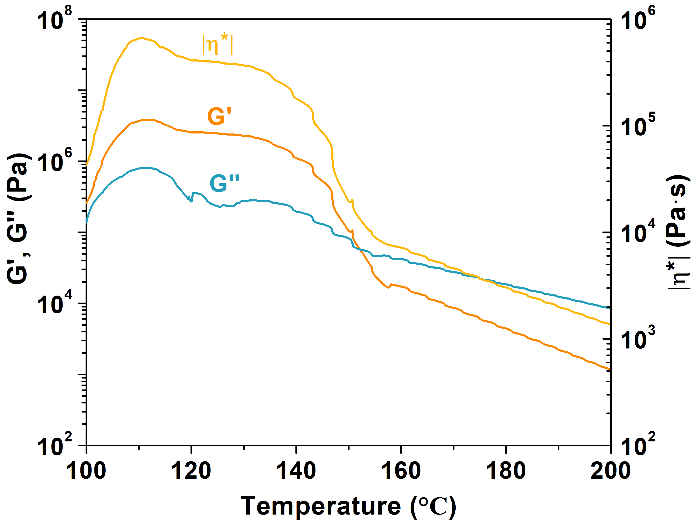
**

**Figure S1**. Oscillatory shear rheological properties of PLLA as a function of temperature: storage modulus (*G’*), loss modulus (*G*’’), and complex viscosity (|*η*^*^|).


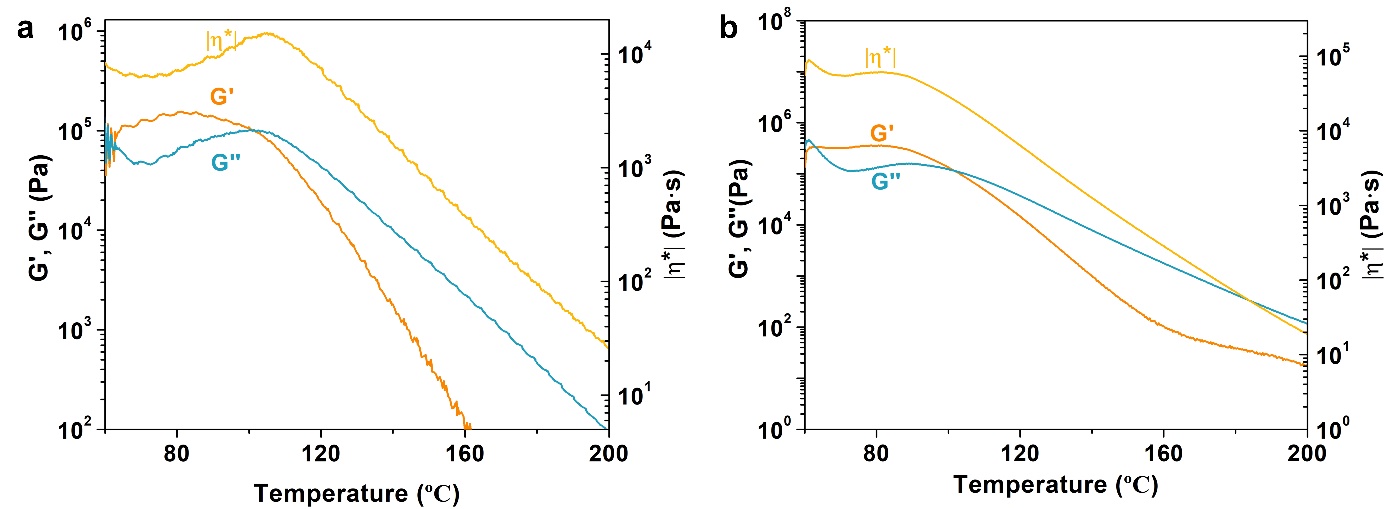


**Figure S2**. Oscillatory shear rheological properties of (a) PLGA 1 and (b) PLGA 2 as a function of temperature: *G*’, *G*’’, and |*η*^*^|.


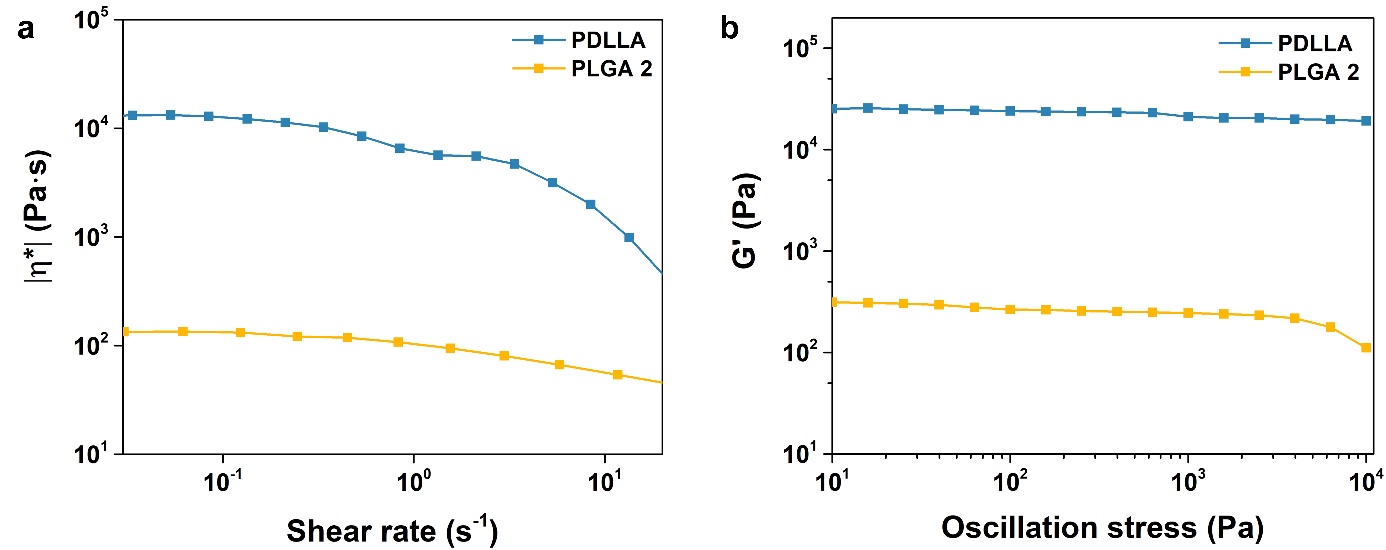


**Figure S3**. (a) Shear thinning behavior of PDLLA and PLGA 2. (b) G’ as a function of the oscillation stress of PDLLA and PLGA 2 at 140 °C.

**Section S2. Optical images of PETG-based thermally drawn fibers.**


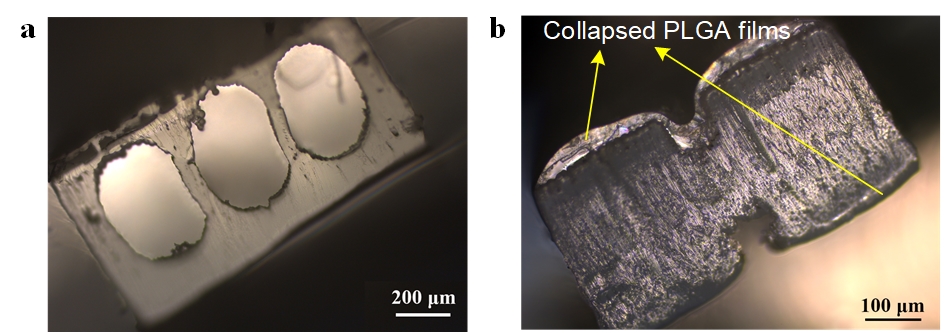


**Figure S4**. Optical images of the PETG-based thermally drawn fibers. (a) Pure PETG fiber with three well-preserved inner channels. (b) Multi-material PETG fiber where the PLGA films collapsed.

**Section S3. Mechanical properties of the SMPFs and compression-molded polymer films.**


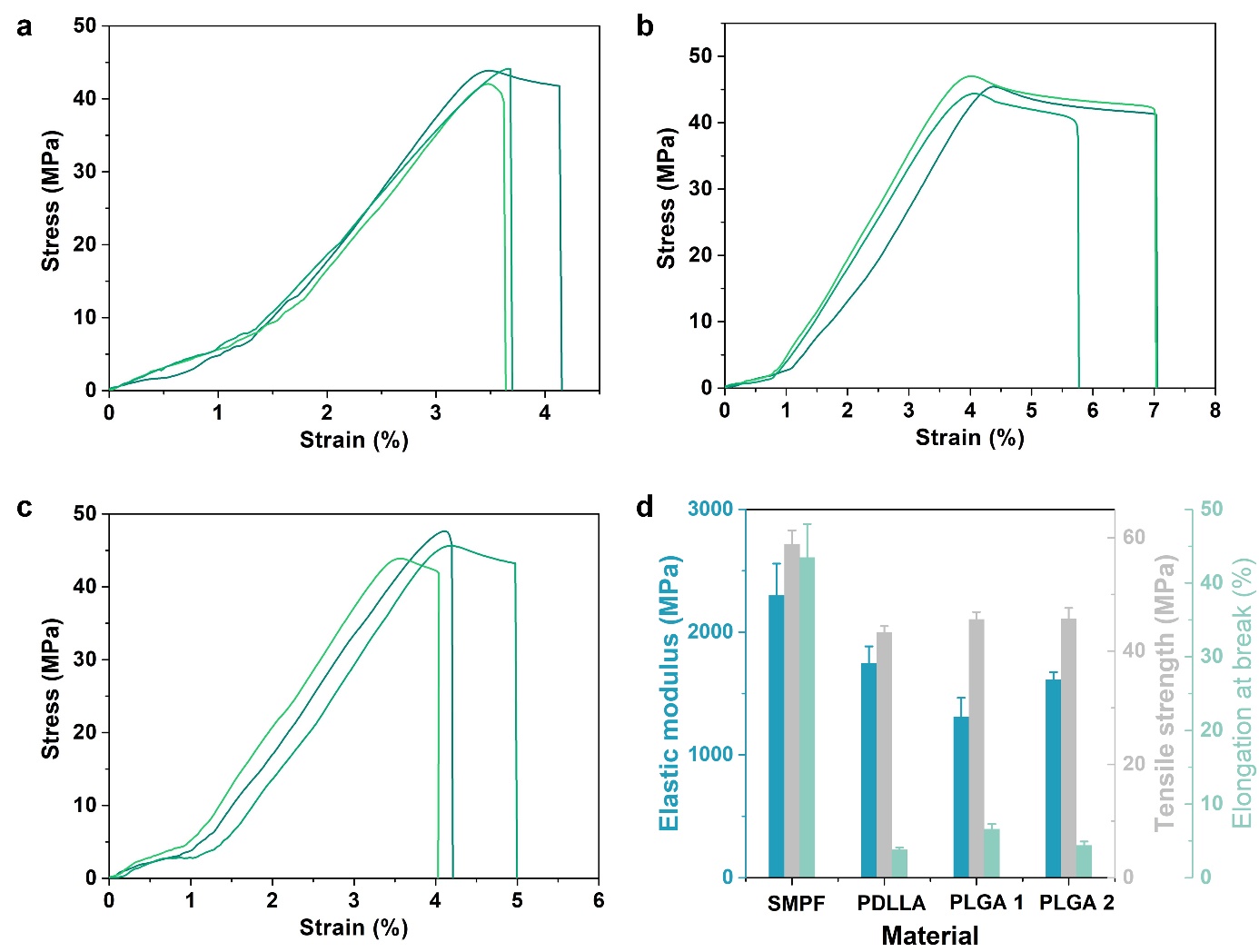


**Figure S5**. Static tensile curves at room temperature for (a) compression-molded PDLLA film, (b) compression-molded PLGA 1 film, and (c) compression-molded PLGA 2 film. The curves correspond to multiple fiber samples. (d) Comparison between the SMPF and compression-molded films.

**Table S1**. Mechanical properties of SMPFs and compression-molded films of PDLLA, PLGA 1, and PLGA 2 at room temperature.

| Material | Technique | ^a^*E* (MPa) | ^b^*σ*_m_ (MPa) | ^c^*ε*_b_ (%) |
| --- | --- | --- | --- | --- |
| SMPF | Thermal drawing | 2301.3 ± 257.9 | 58.9 ± 2.4 | 43.5 ± 4.5 |
| PDLLA | Compression molding | 1747.9 ± 137.1 | 43.3 ± 1.1 | 3.8 ± 0.3 |
| PLGA 1 | Compression molding | 1310.4 ± 155.9 | 45.6 ± 1.3 | 6.6 ± 0.7 |
| PLGA 2 | Compression molding | 1613.9 ± 60.2 | 45.7 ± 1.9 | 4.4 ± 0.5 |

^a^*E*: elastic modulus

^b^*σ*_m_: tensile strength

^c^*ε*_b_: elongation at break

**Section S4. Precise drug loading**

**
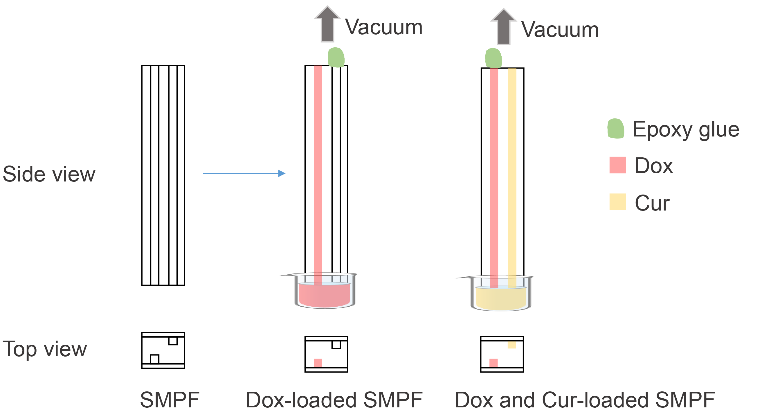
**

**Figure S6**. Schematic diagram of the drug loading process.

Drug loading is caculated by measuring the mass loss in the drug solution (*m*_0_), and the mass gain in the SMPF (*m*_1_):

$$Effiency(\%)=\frac{m_{1}}{m_{0}}\times100\%$$

Since the loading is a purely physical vacuum suction process, drug loss was minimal and mainly due to surface residue. The calcuated drug loading efficiency was around 95%.

**Section S5. Quantitative calibration of Dox and Cur solutions.**


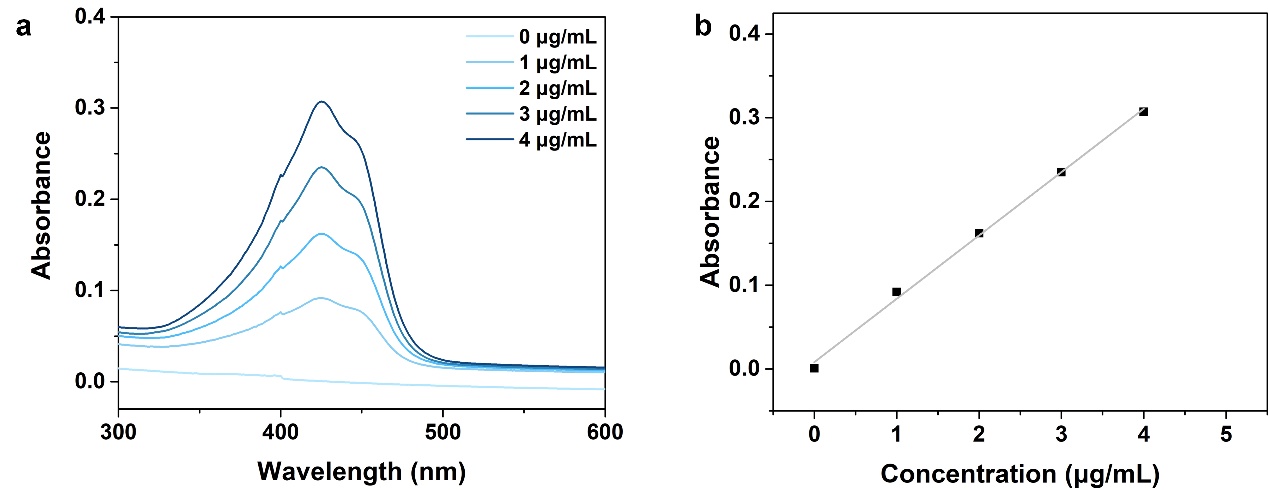


**Figure S7**. Quantitative calibration of Cur solution. (a) UV-Vis absorption spectra at different concentrations. (b) Fitting curve with the equation *A* = 0.0756*C* + 8.13e^-3^ (r^2^ = 0.9988), where *A* is the absorbance and *C* is the Cur concentration.


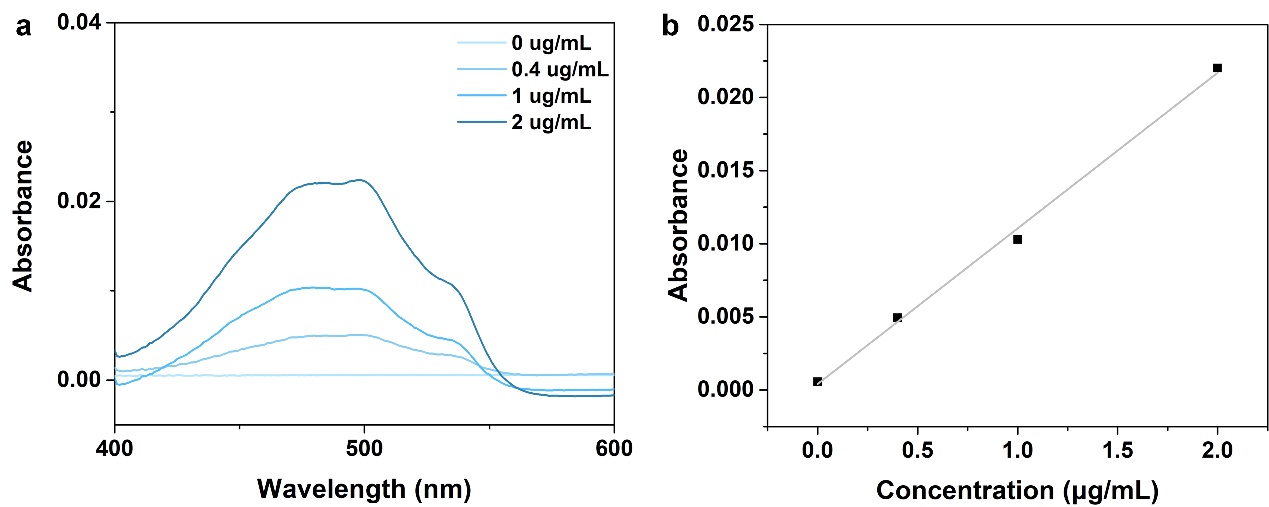


**Figure S8**. Quantitative calibration of Dox solution. (a) UV-Vis absorption spectra at different concentrations. (b) Fitting curve with the equation A = 0.01064C + 4.01e^-4^ (r^2^ = 0.9985), where A is the absorbance and C is the Cur concentration.

**Section S6 Mathematical modeling of drug release kinetics.**

The release profiles of Dox and Cur in Figure 2a were analyzed by using three commonly applied mathematical models including Higuchi, Korsmeyer–Peppas, and Weibull models. The Higuchi model showed a relatively strong correlation with R^2^ values around 0.91, suggesting that diffusion dominated in the release profiles. However, since the Higuchi model assumes a non-degradable matrix and purely diffusion-controlled release, it may not fully apply to PLGA systems which undergo hydrolytic degradation. The semi-empirical Korsmeyer–Peppas model exhibited a fitted exponent slightly greater than 1.0 for Dox release, indicating a Super Case II mechanism in PLGA 1 [1]. This suggest that polymer relaxation and erosion are the dominant factors. For Cur release, the exponent value was between 0.5 to 1.0, indicating a combination of swelling and diffusion mechanism in PLGA 2. Although this model provided a good fit with R^2^ above 0.85, it is limited by its validity up to 60% of the total release. The empirical Weibull model has become popular for describing complex release behavior of PLGA systems. This model showed the best overall fit, with the highest R² values up to 0.93. The fitted β values were greater than 1 for both drugs, indicating a sigmoidal release profile. This suggests that the release process is governed by both diffusion and matrix degradation, which aligns well with the known behavior of PLGA-based delivery systems.

**Table S2**. Comparison of the mathematical models and precisions.

| Model name | Equation | Drug | Fitting parameter | R^2^ |
| --- | --- | --- | --- | --- |
| Higuchi | $f=K\cdot\sqrt{t}$ | Dox | K=0.10375 | 0.92 |
|  |  | Cur | K=0.0702 | 0.91 |
| Korsmeyer- Peppas | $\ln(f)=\ln(K)+n\cdot\ln(t)$ | Dox | n=1.0654 | 0.85 |
|  |  | Cur | n=0.9888 | 0.90 |
| Weibull | $\ln\left[-\ln(1-f)\right]=\ln(\alpha)+\beta\cdot\ln(t)$ | Dox | β=1.4326 | 0.93 |
|  |  | Cur | β=1.1102 | 0.91 |

**Section S7. Characterization of PDLLA, PLGA variants, and PDA nanoparticles**


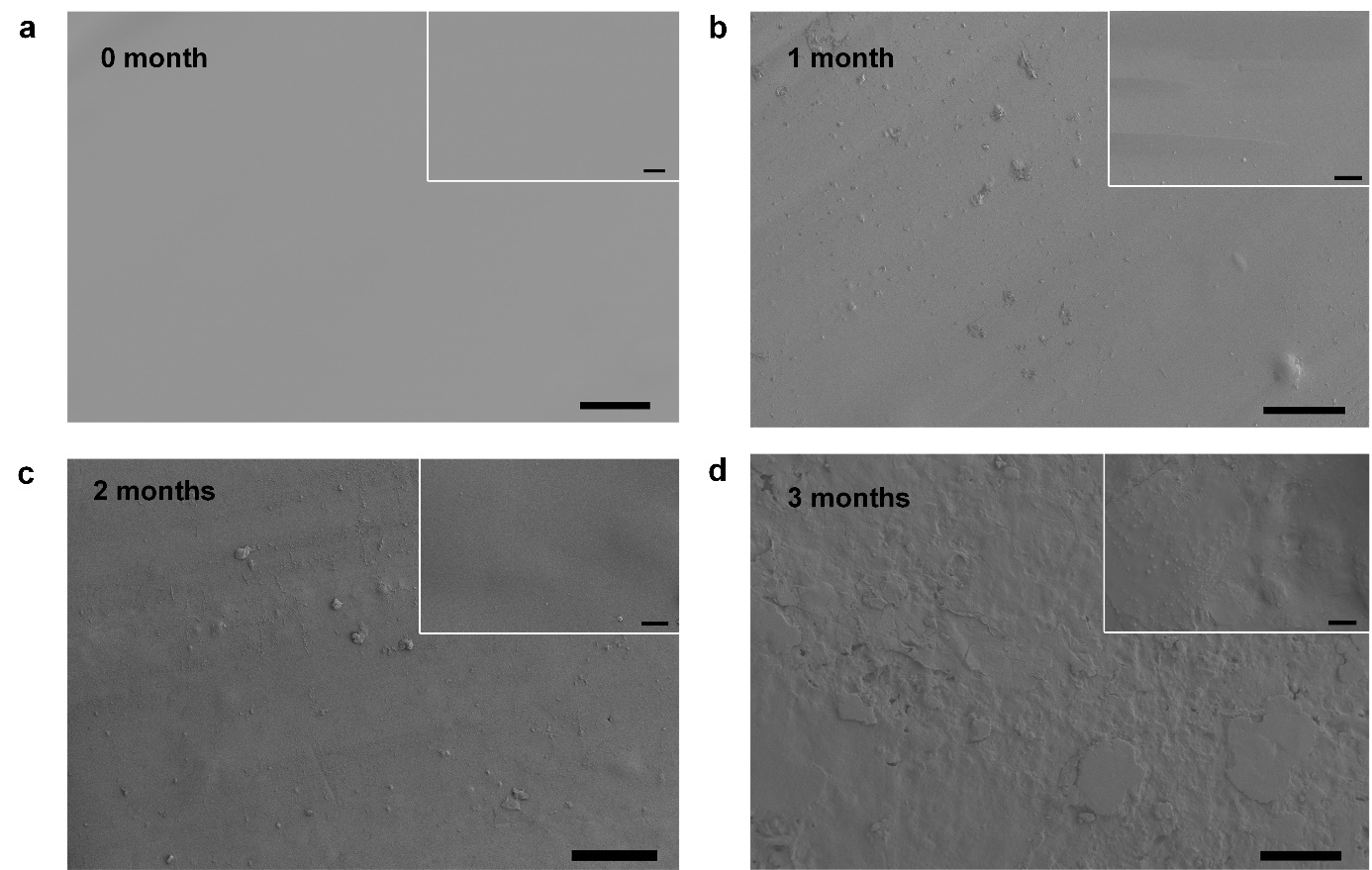


**Figure S9**. Morphological changes of PDLLA during the degradation periods at 1, 2, and 3 months. Scale bars, 100 μm (Inset: 2 μm).


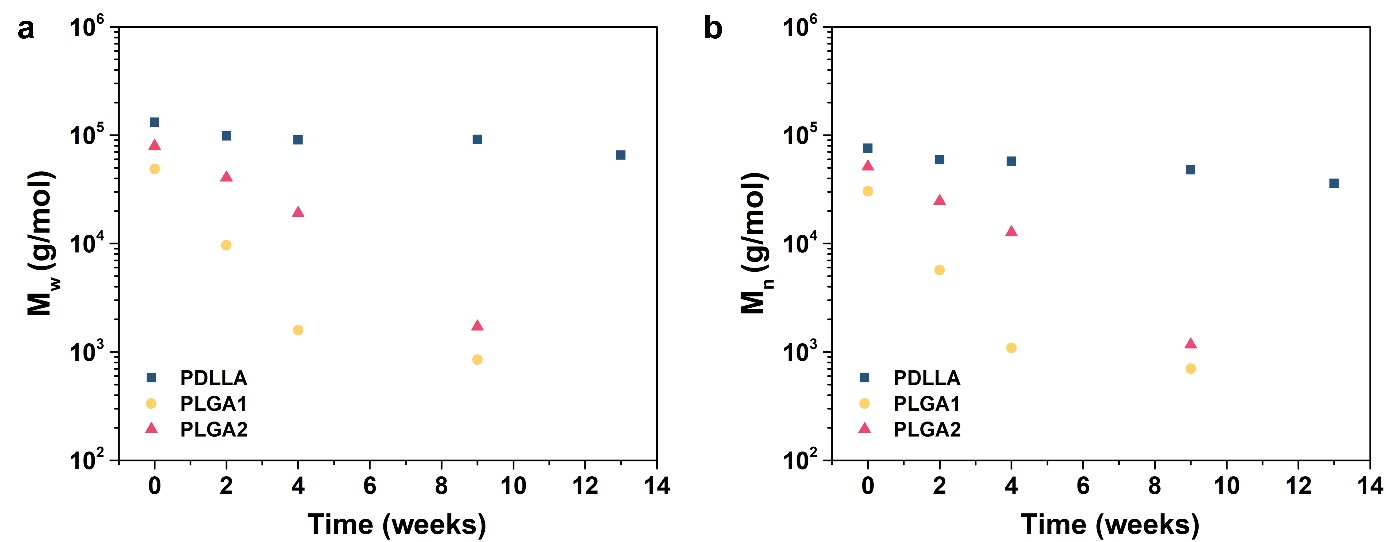


**Figure S10**. Evolution of *M*_w_ and *M*_n_ over time for PDLLA and PLGA variants.


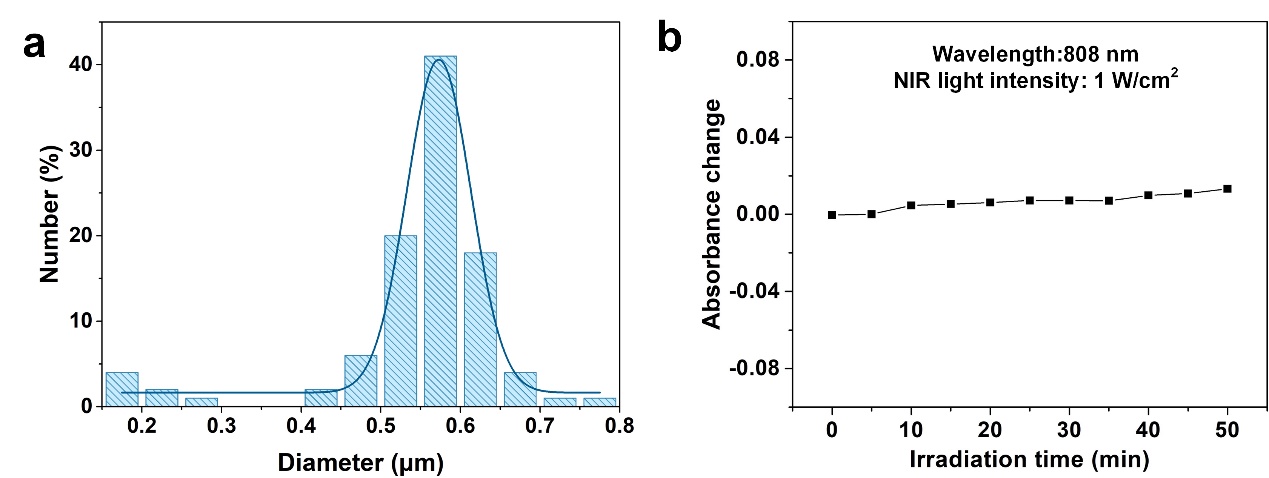


**Figure S11**. Characterization of PDA nanoparticles. (a) Size distribution of the PDA nanoparticles from SEM analysis. (b) Photothermal stability of the PDA@SMPF under NIR light irradiation for 50 minutes (light intensity: 1 W‧cm^−2^).


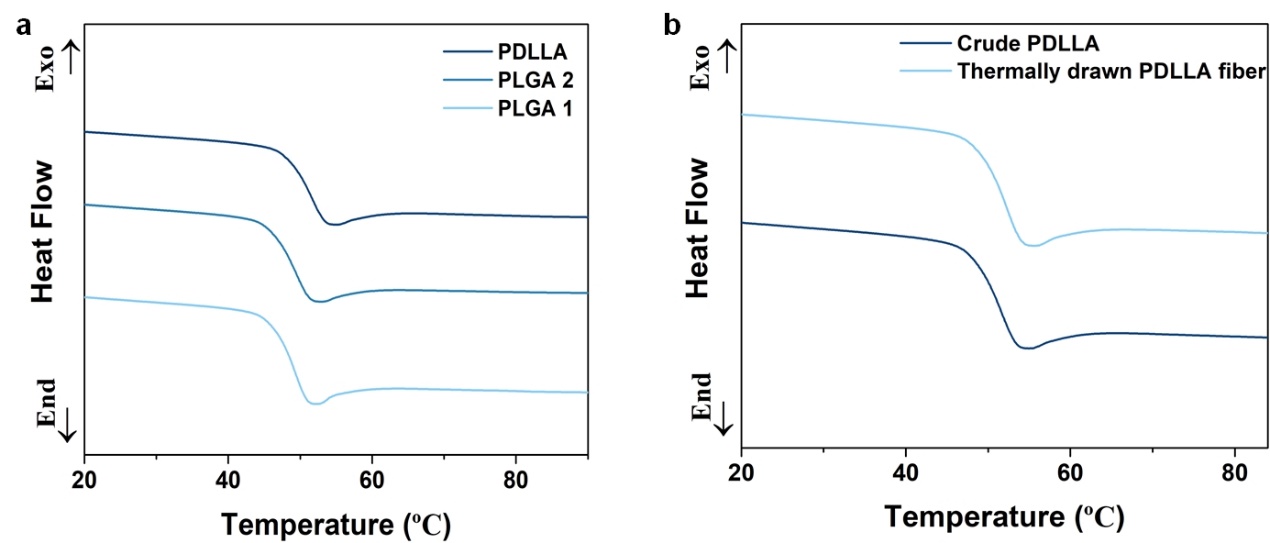


**Figure S12**. Thermal properties of PDLLA and PLGA variatns. (a) DSC curves of the crude PDLLA and PLGA varaiants. (b) DSC curves of the crude PDLLA and thermally drawn pure PDLLA fiber.

**Table S3**. *T*_g_ summary of PDLLA, PLGA 1, and PLGA 2.

| Crude polymer | *T_g_* by DSC (℃) | *T_g_* by DMA (℃) |
| --- | --- | --- |
| PDLLA | 50.2 | 67.5 |
| PLGA 1 | 48.1 | 65.7 |
| PLGA 2 | 48.3 | 66.8 |

**Section S8. Drug release profiles of PDA@SMP fibers.**


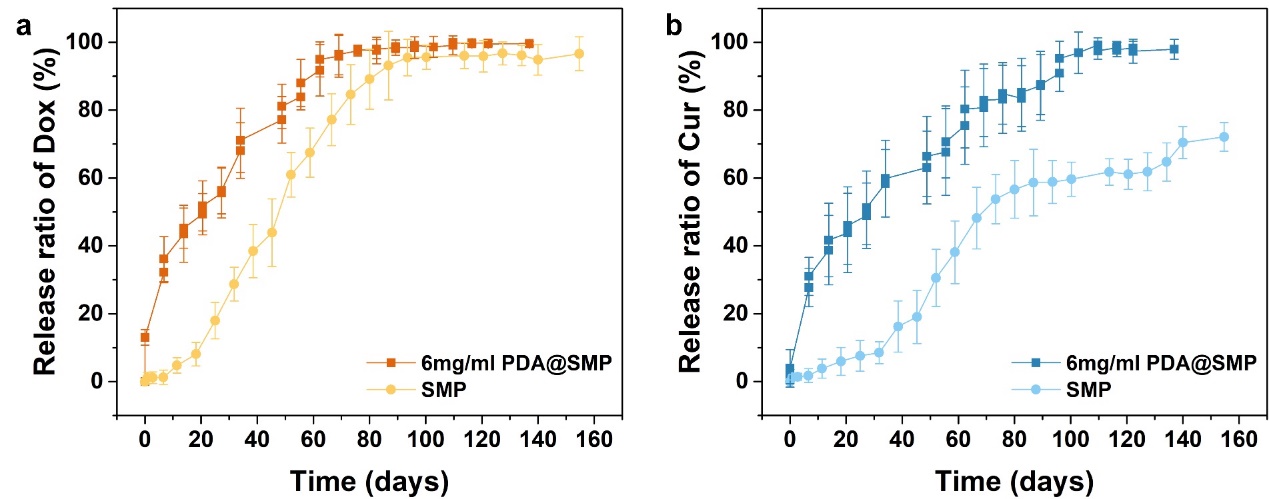


**Figure S13**. Comparison of drug release profiles for (a) Dox and (b) Cur between the 6 mg ml^-1^ PDA@SMPF and the SMPF under intermittent NIR irradiation for 10 min (light intensity: 1 W cm^-2^).


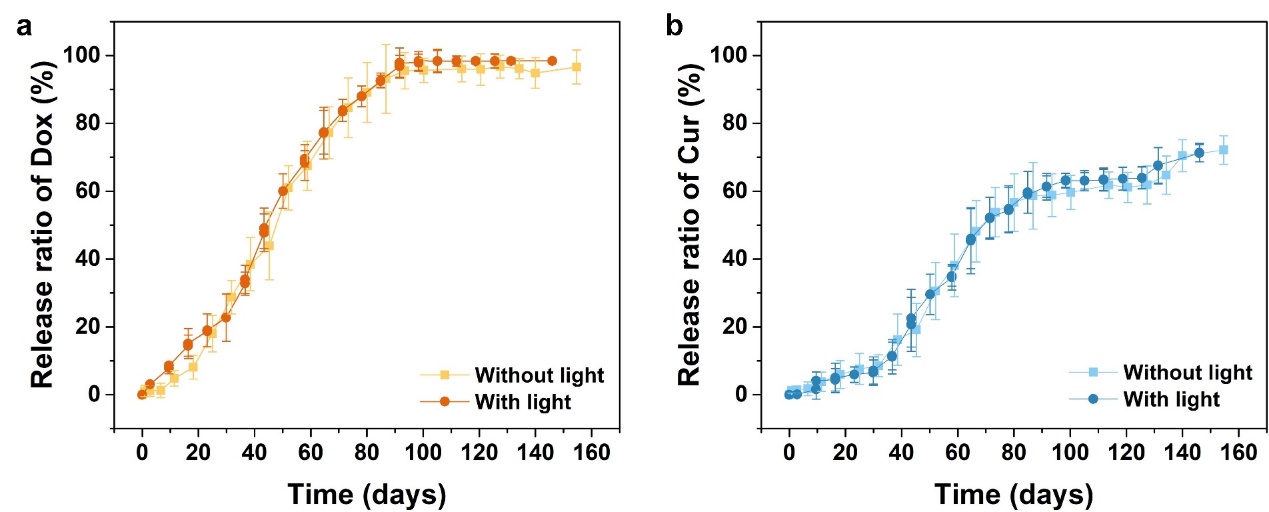


**Figure S14**. Comparison of drug release profiles for (a) Dox and (b) Cur in the SMPF with and without intermittent NIR irradiation for 10 min (light intensity: 1 W cm^-2^).

**Section S9. Adjustable iris for precise controlled localized drug release**


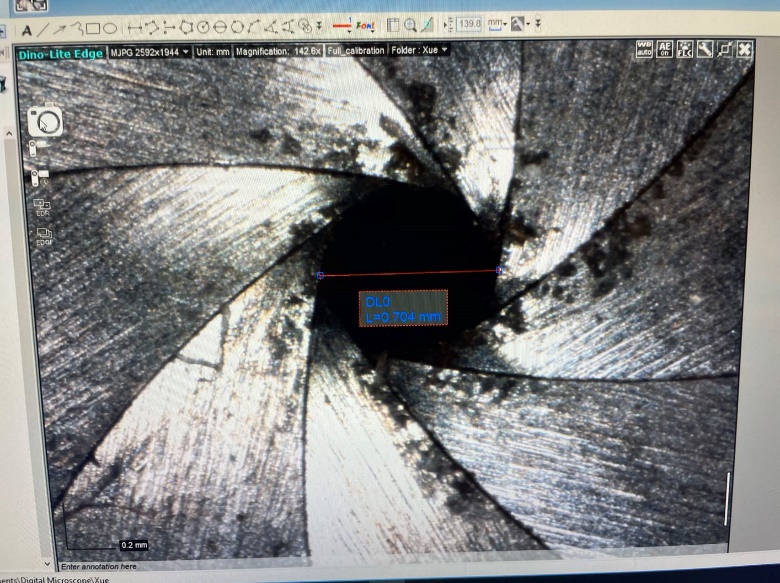


**Figure S15**. Demonstration of an adjustable iris aperture with a diameter of 0.7 mm.

**Section S10. Thermomechanical properties of PDLLA and PLGA variants.**


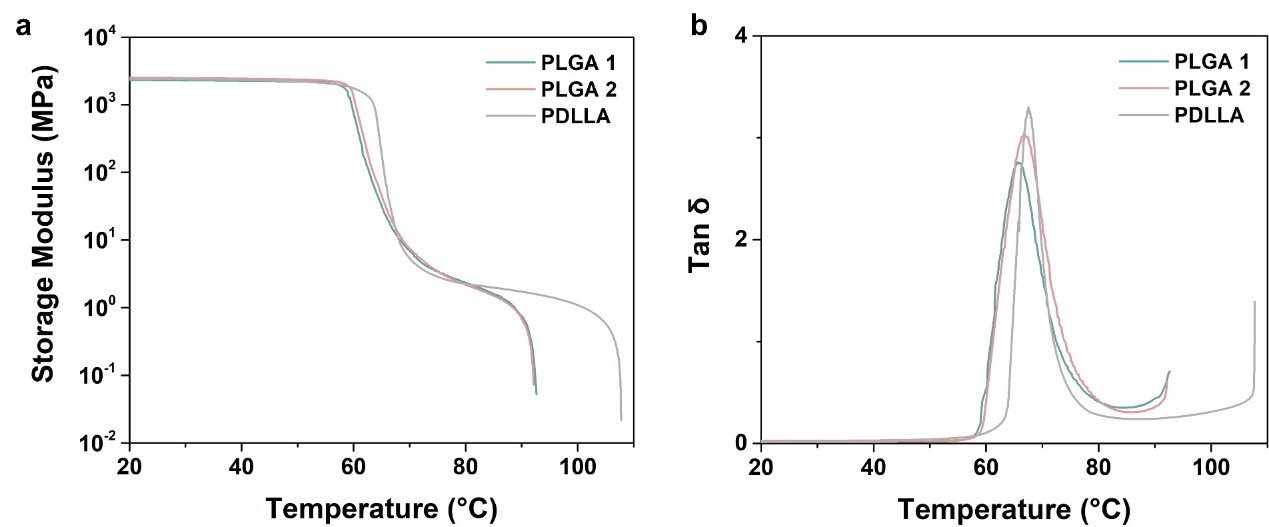


**Figure S16**. Thermomechanical properties of PDLLA and PLGA variants: (a) Storage modulus as a function of temperature. (b) Tan *δ* as a function of temperature.

**Section S11. Stress relaxation and calculation of the activation energy (*E*_a_).**

**
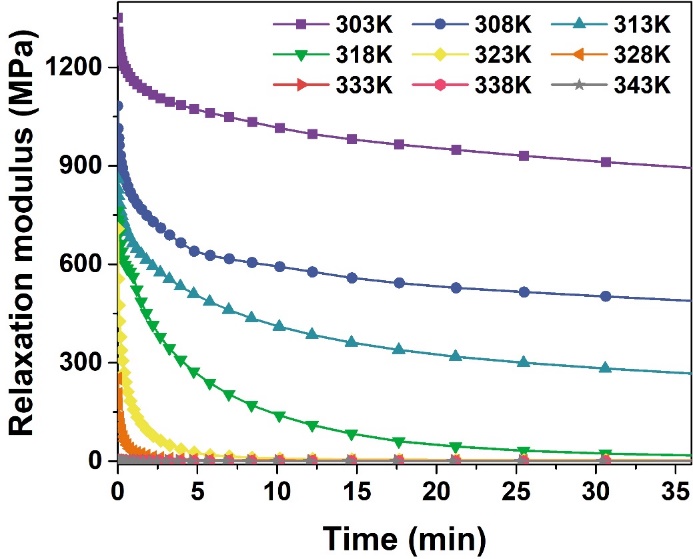
**

**Figure S17**. Temperature-dependent stress relaxation curves of PDLLA.


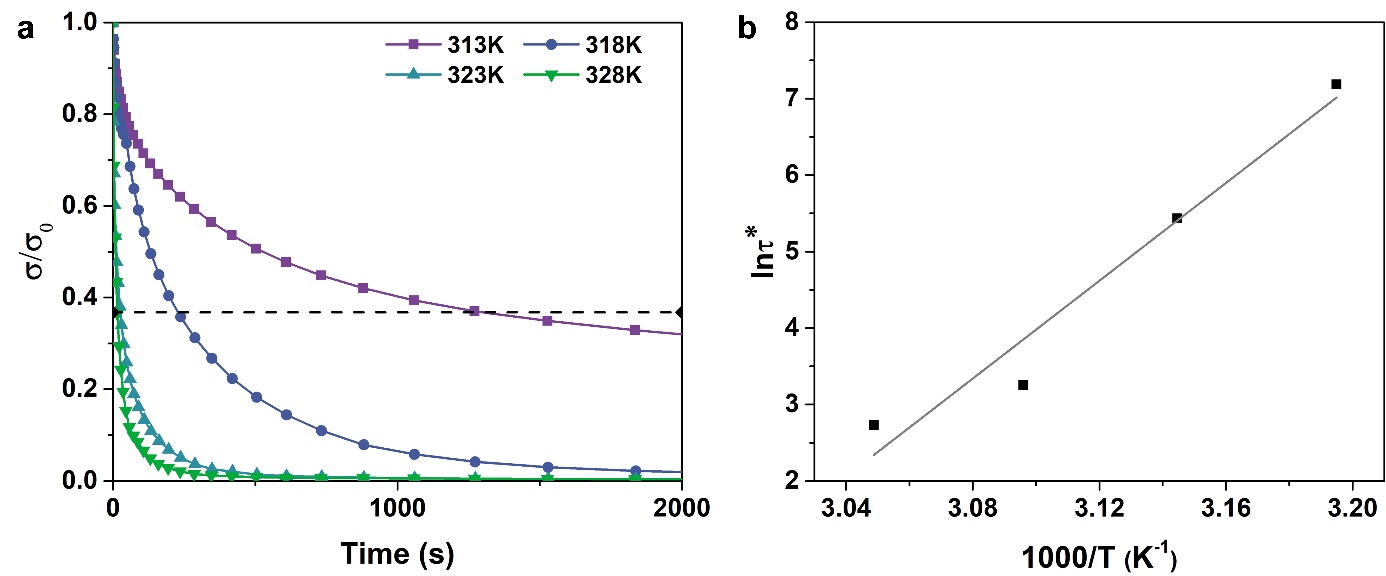


**Figure S18**. (a) Normalized stress relaxation modulus versus time for thermally drawn SMP fibers. (b) Fitting of the stress relaxation time to the Arrhenius’s equation.

From the stress relaxation curves, the relaxation times *τ** could be determined as the time where *σ*/*σ*_0_=e^−1^, which enabled the comparison of relative stress-relaxation rates at different temperatures. Accordingly, by fitting the Arrhenius equation

$$\tau^{*}=\tau_{0}e^{\frac{E_{a}}{RT}}$$

where *T* is the absolute temperature (K), *R* is the molar gas constant (8.314 J K^-1^ mol^-1^), *τ*_0_ is the characteristic relaxation time at y intercept, and *E*_a_ is the activation energy (kJ mol^-1^). By fitting the curve of **Figure S16** into the equation

$$\ln\tau^{*}=\ln\tau_{0}+\frac{E_{a}}{RT}$$

*E*_a_ of PDLLA was calculated to be 265.8 kJ mol^-1^.


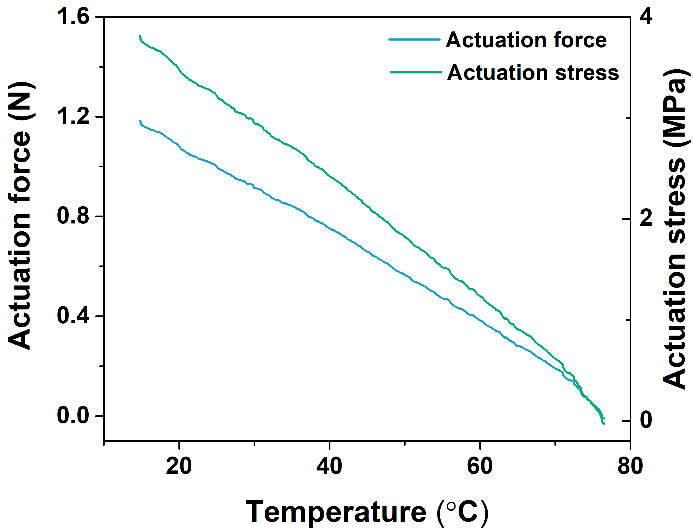


**Figure S19**. Actuation force and actuation stress of the as-fabricated SMPF.

**Section S12. Shape memory properties of the thermally drawn SMPFs.**


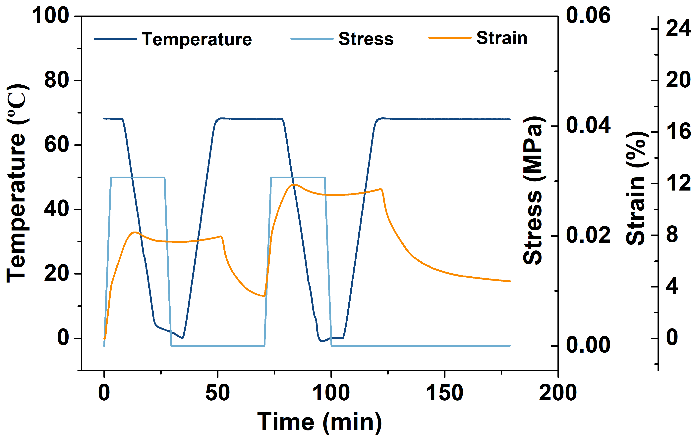


**Figure S20**. Shape memory cycle of the SMPF at a recovery temperature of 68 ℃.


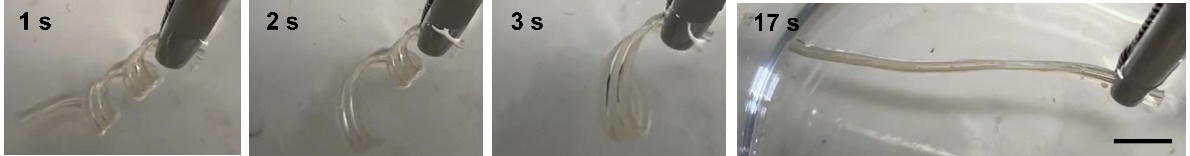


**Figure S21**. Shape programming and recovery of the SMPF from a spiral shape to a flat shape in a water bath at 80 ℃. Scale bar, 5 mm.


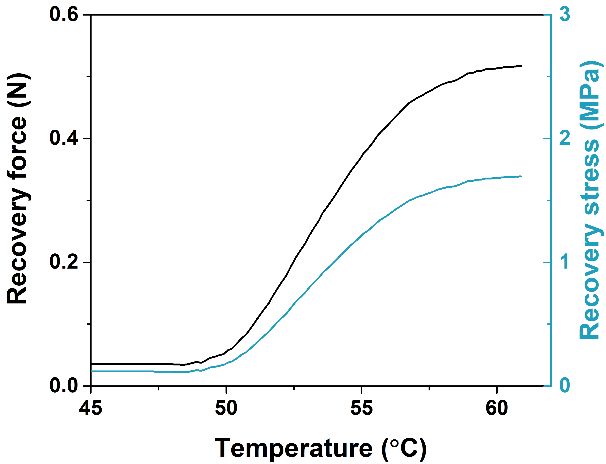


**Figure S22**. Recovery force and stress evoluation during shape recovery. After stress relaxation, the SMPF was programmed into an elongated temporary shape with a stretching ratio of around 100%. The recovery force and stress was monitored by DMA in the iso-strain mode at a temperature ramp of 5 °C min^−1^.


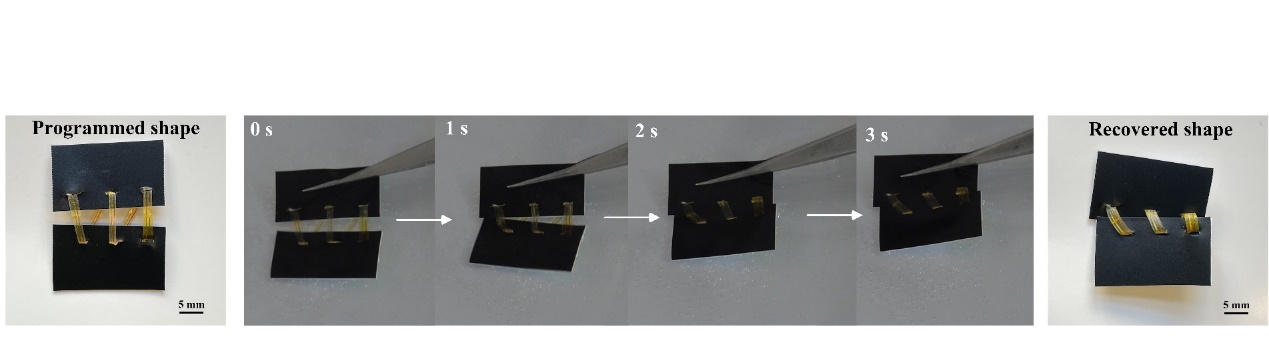


**Figure S23**. Self-tightening behavior of the microstructured SMPF containing Cur and Dox in a water bath at 80 ℃.

**Section S13. Metallic wire–integrated SMPFs.**


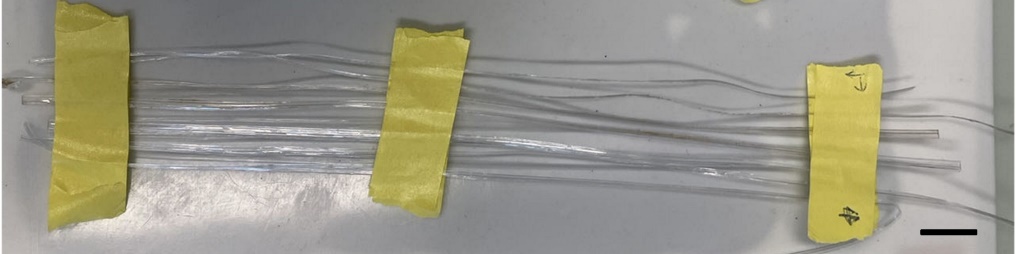


**Figure S24**. Images of the metallic wire–integrated SMPF at a meters-long scale. Scale bar, 5 cm.


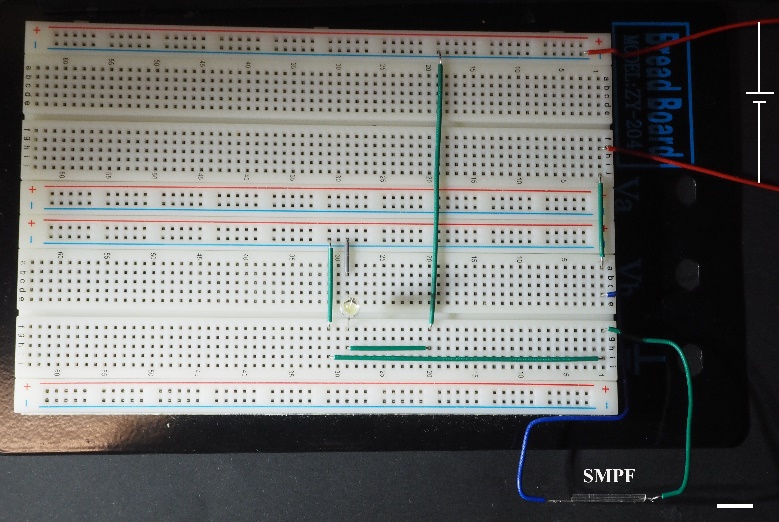


**Figure S25**. Electrical circuit of the metallic wire−integrated SMPF to trigger an LED light. Scale bar, 1 cm.

**Section S14. Fabrication of the SMP preform.**


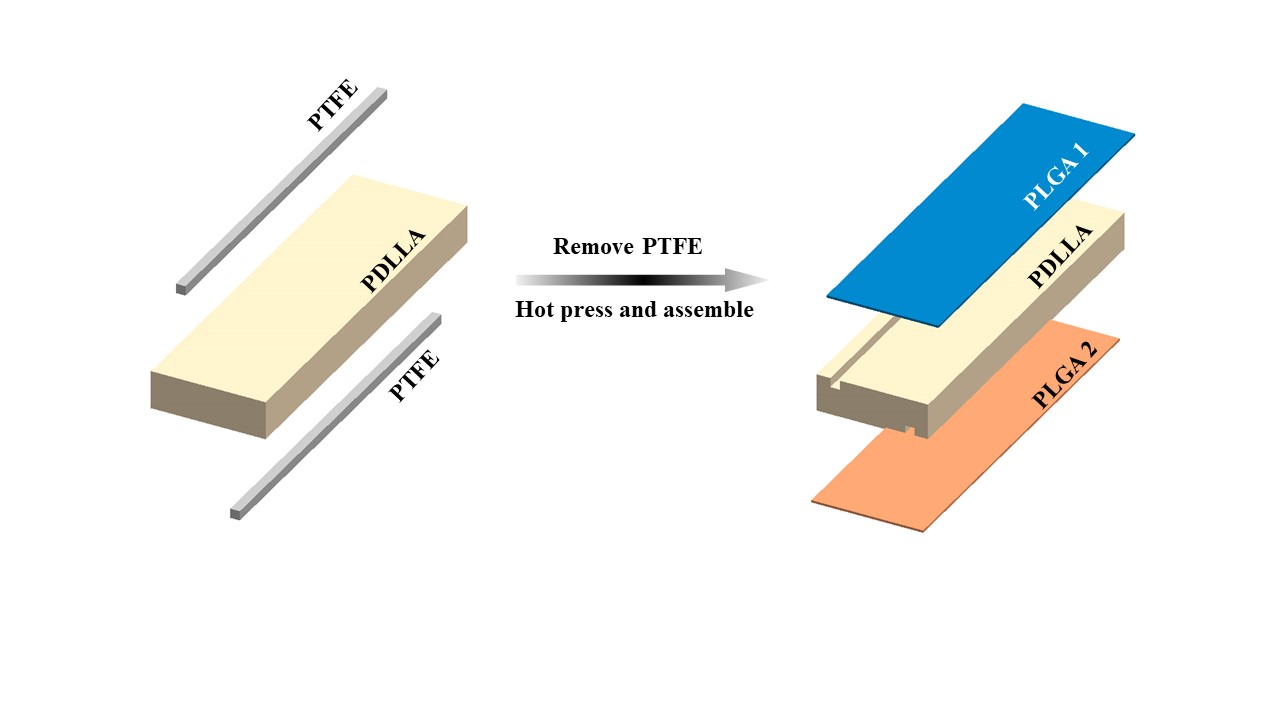


**Figure S26**. Schematic preparation process of the SMP preform.

**Movies.**

Movie S1. Shape recovery process of the multi-material SMPF in a water bath at 80 ℃.

Movie S2. Shape recovery process of the multi-material PDA@SMPF under NIR light (light intensity: 1 W cm^-2^).

Movie S3. Self-tightening behavior of the multi-material SMPF in a water bath at 55 ℃.

**References**

[1] Martín-Camacho UdJ, Rodríguez-Barajas N, Sánchez-Burgos JA, Pérez-Larios A. Weibull β value for the discernment of drug release mechanism of PLGA particles. Int. J. Pharm. 2023, 640: 123017.

1. ^*^ Corresponding author. Fabien Sorin: [fabien.sorin@epfl.ch](mailto:fabien.sorin@epfl.ch) [↑](#footnote-ref-1)
